# Redox-exercise crosstalk in neurodegenerative diseases: mechanistic perspectives on antioxidant interactions with Nrf2-BDNF signaling, autophagy, and mitochondrial plasticity

**DOI:** 10.3389/fnut.2026.1850414

**Published:** 2026-08-13

**Authors:** Lifu Liu, Yunpeng Zhao

**Affiliations:** 1 Ministry of Sports and National Defense Education, Chongqing Polytechnic University of Electronic Technology, Chongqing, China; 2 Department of Physical Education, Hainan Medical University, Haikou, China

**Keywords:** autophagy, exercise, flavonoid antioxidants, learning, memory, neurodegenerative diseases, trace elements, vitamin

## Abstract

Neurodegenerative diseases (NDDs), including Alzheimer’s disease and Parkinson’s disease, are characterized by progressive neuronal loss driven by oxidative stress, mitochondrial dysfunction, and impaired cellular homeostasis. Emerging research highlights a complex interplay between physical exercise and antioxidant mechanisms in the regulation of redox balance and neuroprotection. This review evaluates the integrative effects of exercise and antioxidant interventions on molecular pathways involved in NDDs, emphasizing mechanistic and translational findings from both preclinical and clinical studies. This narrative review was informed by a structured literature search conducted in PubMed, Scopus, and Web of Science, focusing on studies investigating exercise, antioxidants, and NDDs. Current studies indicate that vitamins, trace elements (particularly selenium and zinc), endogenous antioxidant systems, and flavonoids may interact with exercise through partially overlapping mechanisms that may influence neurodegenerative processes. These interventions influence several interconnected molecular pathways, including redox-sensitive Nrf2 signaling, BDNF-mediated neuroplasticity, autophagy, mitochondrial plasticity, and gut-brain axis communication. Exercise-induced activation of redox-sensitive pathways enhances endogenous antioxidant defenses. Under appropriate conditions, antioxidant supplementation may complement these adaptations by limiting excessive oxidative stress and supporting cellular metabolism. Available data suggest that combined exercise-antioxidant interventions may provide additional neuroprotective benefits in some experimental settings; however, these effects are highly context-dependent and are not consistently superior to exercise alone. Furthermore, exercise-antioxidant interactions may influence epigenetic regulation and neurotrophic signaling, thereby contributing to neuroprotection. However, the literature remains limited by substantial heterogeneity in intervention protocols, antioxidant type, dosage and timing, exercise characteristics, disease stage, and the scarcity of large-scale clinical trials. Future research should prioritize clinical validation and clarify how antioxidant type, dosage, timing, and exercise parameters influence adaptive redox signaling, hormetic responses, and neuroprotective outcomes.

## 1 Introduction

Neurodegenerative disorders (NDDs) represent a growing global health challenge, with Alzheimer’s disease (AD) and Parkinson’s disease (PD) being the most prevalent forms. According to recent World Health Organization and Global Burden of Disease reports, the prevalence of dementia and Parkinsonian disorders continues to rise substantially due to population aging, imposing major socioeconomic and healthcare burdens worldwide. It is estimated that more than 55 million individuals currently live with dementia globally, and this number is projected to nearly triple by 2050 (1, 2). AD is characterized by progressive cognitive decline, memory deficits, and the pathological accumulation of amyloid-β (Aβ) plaques alongside tau neurofibrillary tangles, whereas PD is chiefly characterized by motor impairments, the loss of dopaminergic neurons within the substantia nigra, and the formation of Lewy bodies comprised of *α*-synuclein aggregates. Despite their distinct clinical presentations, both conditions are underpinned by shared molecular and cellular pathways, including mitochondrial dysfunction, persistent inflammation, compromised proteostasis, and heightened oxidative stress, which facilitate neuronal degeneration and functional impairment (3, 4). Oxidative stress plays a central role in the development and progression of NDDs. This condition results from an imbalance between the generation of reactive oxygen species (ROS) and the efficacy of intrinsic antioxidant defense mechanisms to mitigate their effects. Neurons exhibit heightened susceptibility to oxidative injury owing to their elevated metabolic demands, substantial lipid composition, and comparatively diminished antioxidant capacity. Excessive ROS can damage lipids, proteins, and DNA, culminating in synaptic dysfunction, compromised neuronal signaling, and ultimately, cellular demise. In the contexts of AD and PD, oxidative stress is intricately associated with mitochondrial dysfunction, wherein disrupted electron transport chain functionality engenders an upsurge in ROS production, thereby exacerbating cellular impairment. Oxidative stress plays a significant role in the misfolding and aggregation of proteins, notably Aβ and *α*-synuclein, thereby establishing a deleterious feedback loop that hastens neurodegeneration (5–7).

The nuclear factor erythroid 2–related factor 2 (Nrf2) pathway is a key regulator of cellular redox homeostasis (8). Nrf2 regulates the transcription of a diverse array of antioxidant and cytoprotective genes, encompassing those that participate in glutathione biosynthesis, detoxification processes, and mitochondrial functionality (9). Nevertheless, in the context of NDDs, Nrf2 signaling frequently becomes dysregulated, diminishing the capacity of neurons to effectively mitigate oxidative stress. Likewise, perturbations in autophagy, an essential cellular mechanism tasked with the degradation and recycling of impaired organelles and misfolded proteins, further exacerbate the progression of these diseases. Compromised autophagy results in the accumulation of deleterious protein aggregates and dysfunctional mitochondria, thereby intensifying oxidative damage and subsequent neuronal degeneration (10, 11). Emerging evidence also highlights the involvement of epigenetic regulators, neurotrophic signaling pathways, and gut-brain communication networks in the pathogenesis and progression of NDDs (12, 13). Alterations in gut microbiota composition, intestinal barrier permeability, and microbiota-derived metabolites have been associated with neuroinflammation, oxidative stress, and impaired neuronal signaling in both AD and PD (14, 15). Exercise and antioxidant interventions may influence gut microbiota diversity and gut-brain axis signaling, thereby contributing to neuroprotection and cognitive resilience (16, 17). Current pharmacological interventions for AD and PD predominantly serve a symptomatic role and fail to effectively arrest or reverse the trajectory of disease progression. These constraints underscore the pressing necessity for innovative therapeutic approaches aimed at the fundamental molecular mechanisms underlying these pathologies. Numerous pharmacological agents are linked to adverse effects, diminishing efficacy over time, and an inability to simultaneously address the multifaceted disease processes, including oxidative stress, inflammation, and mitochondrial dysfunction (18, 19).

Among non-pharmacological strategies, physical exercise has received considerable attention because of its neuroprotective effects. Regular exercise has been shown to enhance antioxidant defense mechanisms, facilitate mitochondrial biogenesis, promote neurogenesis, and modulate synaptic plasticity. Notably, physical exercise has the capacity to activate the Nrf2 signaling pathway, thereby elevating the expression of endogenous antioxidants and mitigating oxidative stress. Regular physical activity has been shown to enhance cognitive functions, memory retention, and learning capabilities, in part through the upregulation of neurotrophic factors such as brain-derived neurotrophic factor (BDNF) and the promotion of hippocampal plasticity (13, 20). The term “Nrf2-BDNF axis” is used in the present review as a conceptual framework describing the functional interplay between redox-sensitive Nrf2 signaling and BDNF-mediated neuroplastic pathways rather than a single linear signaling cascade. Emerging evidence suggests that exercise-induced redox signaling may coordinately regulate Nrf2 activation and BDNF expression through interconnected mechanisms involving ROS-mediated hormetic responses, cAMP response element-binding protein (CREB) activation, mitochondrial adaptations, and modulation of neuroinflammatory pathways (21, 22). Activation of Nrf2 may indirectly preserve BDNF signaling by reducing oxidative stress, attenuating neuroinflammation, and maintaining mitochondrial homeostasis, whereas BDNF contributes to neuronal survival, synaptic plasticity, and mitochondrial function through downstream signaling pathways such as phosphoinositide 3-kinase (PI3K)/protein kinase B (PKB or Akt) and ERK/CREB (23, 24). Therefore, the proposed Nrf2-BDNF axis should be viewed as a dynamic and context-dependent interaction network that may contribute to exercise-induced neuroprotection in neurodegenerative diseases rather than as a universally established unidirectional pathway. In addition to the benefits of exercise, dietary antioxidants including vitamins, trace elements, endogenous antioxidants, and flavonoids, serve vital functions in the preservation of redox homeostasis and the safeguarding of neuronal integrity. These bioactive compounds have the capacity to directly neutralize ROS, modulate cellular signaling pathways such as the Nrf2, and enhance mitochondrial functionality. In the present review, the term “mitochondrial plasticity” refers to the ability of mitochondria to flexibly adapt their structure, function, and turnover in response to physiological or pathological stimuli (25, 26). This concept encompasses several interconnected processes, including mitochondrial biogenesis, fission and fusion dynamics, mitophagy, bioenergetic flexibility, and mitochondrial stress responses (27, 28). These adaptive processes are essential for maintaining cellular energy homeostasis, redox balance, and neuronal survival, particularly within the energetically demanding environment of the central nervous system (26). Impairments in mitochondrial plasticity have increasingly been associated with the pathogenesis and progression of neurodegenerative diseases, whereas exercise and redox-sensitive interventions may enhance mitochondrial adaptability and resilience (25, 28). Flavonoids have demonstrated the ability to traverse the blood–brain barrier, thereby exerting neuroprotective effects through the modulation of synaptic signaling, attenuation of inflammation, and enhancement of cognitive capabilities. Collectively, current evidence suggests that exercise and antioxidant interventions may interact through shared redox-sensitive pathways, although the magnitude of these effects appears to depend on intervention-specific factors such as antioxidant type, dosage, and exercise characteristics (29–31).

Although growing evidence suggests that exercise and antioxidant interventions may exert complementary neuroprotective effects, the interaction between exogenous antioxidants and exercise-induced adaptive signaling remains controversial (32–34). Recent studies addressing the “antioxidant paradox” indicate that excessive antioxidant supplementation may attenuate exercise-mediated mitohormesis, mitochondrial biogenesis, and endogenous antioxidant responses by suppressing physiologically important ROS signaling (32–35). Therefore, the effectiveness of combined interventions likely depends on antioxidant dosage, timing of administration, exercise modality and intensity, disease stage, and treatment duration. Importantly, the suppression of exercise-induced adaptive signaling by excessive antioxidant exposure may partially explain the neutral or inconsistent findings reported in some studies. This interaction, often referred to as redox–exercise crosstalk, has the potential to optimize cellular resilience, enhance mitochondrial functionality, regulate autophagy, influence epigenetic modifications, and restore redox equilibrium in NDDs (12, 13). Although numerous studies have examined exercise and antioxidant interventions separately, the broader mechanistic relationships linking redox signaling, neurotrophic regulation, mitochondrial plasticity, autophagy, and gut-brain communication remain incompletely integrated across the literature. Accordingly, the objective of this review is to critically examine the integrative interactions between exercise-induced redox signaling and antioxidant systems in NDDs, with particular emphasis on Nrf2-BDNF signaling, mitochondrial plasticity, autophagy, gut-brain axis communication, neurotrophic regulation, and epigenetic mechanisms. This review evaluates the translational potential and current limitations of combined exercise-antioxidant interventions in AD and PD. The novelty of this review lies in its integrative framework, emphasizing the synergistic potential inherent in the conjunction of lifestyle modifications with nutritional strategies aimed at targeting multiple interrelated pathways implicated in neurodegeneration. By clarifying the mechanistic basis of redox–exercise interactions, this review aims to identify potential opportunities for preventive and therapeutic interventions in AD and PD.

## 2 Methods and search strategy

This narrative review was informed by a structured literature search conducted in PubMed, Scopus, and Web of Science. Articles published in English up to December 2025 were identified using combinations of keywords including “exercise,” “physical activity,” “antioxidants,” “oxidative stress,” “neurodegenerative diseases,” “Alzheimer’s disease,” “Parkinson’s disease,” “Nrf2,” “BDNF,” “autophagy,” “mitochondrial dysfunction,” and “gut-brain axis.” Studies were selected based on their relevance to the molecular mechanisms and translational implications of exercise and antioxidant interventions in NDDs. Both preclinical and clinical evidence were considered, including experimental animal studies, cell culture investigations, observational studies, and clinical trials. Articles focusing on unrelated neurological conditions, non-English publications, conference abstracts without full-text availability, and studies lacking mechanistic or translational relevance were excluded. Particular emphasis was placed on recent high-quality studies as well as seminal investigations that have shaped current understanding of redox signaling, neuroprotection, mitochondrial function, autophagy, neurotrophic signaling, and exercise-antioxidant interactions. Given the narrative nature of this review, formal systematic-review procedures, quantitative synthesis, and risk-of-bias assessments were not performed. The selected literature was critically synthesized to identify common mechanistic themes, sources of heterogeneity, and potential translational implications regarding the interaction between exercise and antioxidant systems in AD and PD.

## 3 Oxidative stress and redox imbalance in NDDs

Oxidative stress is a central feature of NDDs, including AD and PD, reflecting an imbalance between the production of ROS and the efficacy of antioxidant defenses. Neurons exhibit heightened vulnerability due to their elevated oxygen utilization, the presence of substantial polyunsaturated fatty acids, and their restricted capacity for regeneration. The disruption of redox equilibrium interferes with cellular signaling pathways, inflicts damage on biomolecular structures, and hinders synaptic plasticity, ultimately leading to declines in cognitive and motor functions. The Nrf2 pathway, recognized as a principal regulator of antioxidant mechanisms, is frequently found to be dysregulated in NDDs, thereby intensifying oxidative damage. Prolonged oxidative stress also engages with neuroinflammatory processes and protein aggregation, establishing a pathogenic cycle that accelerates the advancement of the disease. Consequently, comprehending the origins and repercussions of ROS is imperative for the formulation of targeted therapeutic interventions aimed at re-establishing redox homeostasis (10, 36).

Emerging empirical data further indicates that systemic oxidative stress may modulate gut-brain communication pathways via alterations in the integrity of the intestinal barrier, the composition of gut microbiota, and the production of microbiota-derived metabolites. Enhanced intestinal permeability and gut dysbiosis have been correlated with exacerbated neuroinflammatory responses, mitochondrial dysfunction, and compromised neuronal signaling in NDDs conditions, including AD and PD. Physical exercise and antioxidant interventions may partially mitigate these adverse effects by fine-tuning redox homeostasis, preserving the diversity of gut microbiota, and diminishing inflammatory signaling along the gut-brain axis (14, 15, 37). Although excessive ROS production contributes to oxidative damage and neurodegeneration, physiological levels of ROS also serve important signaling functions. Emerging evidence supports the concept of redox hormesis, whereby transient increases in ROS activate adaptive pathways involved in antioxidant defense, mitochondrial biogenesis, autophagy, and neuroplasticity. This dual role of ROS provides a mechanistic basis for the antioxidant paradox discussed throughout this review and highlights the importance of maintaining redox balance rather than indiscriminate suppression of oxidative processes.

### 3.1 Sources of ROS in the brain

The brain is particularly susceptible to oxidative stress as a consequence of numerous intrinsic mechanisms responsible for the generation of ROS. Mitochondria serve as the predominant source, wherein electron leakage from the electron transport chain results in the formation of superoxide during the process of oxidative phosphorylation. Various enzymatic systems, including NADPH oxidases, xanthine oxidase, and monoamine oxidase, play a substantial role in the production of ROS within both neuronal and glial cells. The metabolism of neurotransmitters, particularly the oxidation of dopamine in the context of PD, exacerbates the oxidative load by facilitating the generation of hydrogen peroxide and reactive quinones. Additionally, activated microglia are known to generate excessive amounts of ROS and reactive nitrogen species in response to neuroinflammatory processes, thereby exacerbating neuronal injury. Environmental factors, such as toxic substances and heavy metals, may further augment ROS production. These varied sources overwhelm the capacity of endogenous antioxidant mechanisms, resulting in oxidative damage and playing a significant role in the pathogenesis of NDDs, including AD (38, 39).

### 3.2 Lipid peroxidation, protein oxidation, DNA damage

Oxidative stress precipitates profound damage to cellular macromolecules, encompassing lipids, proteins, and DNA, which are essential for the maintenance of neuronal integrity. Lipid peroxidation proves especially deleterious in the cerebral context due to the prevalence of polyunsaturated fatty acids, culminating in the synthesis of harmful aldehydes such as malondialdehyde (MDA) and 4-hydroxynonenal. These metabolic byproducts disrupt the structural integrity of membranes, hinder ion gradients, and undermine synaptic functionality. The oxidation of proteins results in structural alterations, inactivation of enzymes, and heightened vulnerability to aggregation, thereby contributing to characteristic pathologies such as Aβ plaques in AD and *α*-synuclein aggregates in PD. Oxidative damage to DNA, manifesting as strand breaks and base modifications, influences both nuclear and mitochondrial genomes, thereby compromising gene expression and cellular repair mechanisms. In aggregate, these oxidative modifications are pivotal in the etiology of neuronal dysfunction and degeneration (40, 41).

### 3.3 Mitochondrial dysfunction

Mitochondrial dysfunction constitutes a pivotal factor in the pathogenesis of oxidative stress and neurodegenerative processes. Mitochondria serve as the fundamental generators of adenosine triphosphate (ATP) within cells, while concurrently acting as significant sources of ROS when their operational capacity is compromised. In the context of NDDs, such as AD and PD, perturbations within the electron transport chain precipitate a decline in ATP synthesis and an increase in electron leakage, culminating in the overproduction of ROS. Compromised mitochondria additionally liberate pro-apoptotic factors, instigating pathways that lead to neuronal cell demise. Disruptions in mitochondrial dynamics, encompassing aberrations in fission, fusion, and mitophagy, further intensify the aggregation of dysfunctional organelles. Mutations in mitochondrial DNA and oxidative insults undermine respiratory performance and cellular robustness. The intricate relationship between mitochondrial dysfunction and oxidative stress engenders a self-reinforcing loop that accelerates neuronal damage, underscoring the importance of mitochondria as pivotal therapeutic targets in the realm of NDDs (42, 43).

### 3.4 Autophagy-related molecular mechanisms in neuroprotection

Autophagy constitutes a fundamental cellular quality-control mechanism that is significantly involved in the neuroprotective effects associated with physical exercise and antioxidant supplementation in the context of NDDs. At the molecular level, the regulation of autophagy is predominantly mediated by the mechanistic target of rapamycin (mTOR) signaling pathway, which functions as a principal suppressor of autophagy initiation under conditions characterized by nutrient abundance (44, 45). Empirical evidence indicates that both physical activity and bioactive compounds effectively diminish mTOR activity, consequently facilitating the activation of ULK1, which is recognized as a pivotal initiator of autophagosome formation (45, 46). The subsequent recruitment of the Beclin-1 complex is instrumental in vesicle nucleation, while the conversion of LC3-I to LC3-II is critical for the elongation and maturation of the autophagosome membrane (46, 47). An increase in LC3-II expression is thereby widely regarded as a definitive indicator of enhanced autophagic flux (47). By modulating these molecular components, the synergistic application of exercise and antioxidant interventions may augment the clearance of aggregated proteins, such as Aβ and phosphorylated tau, thereby fostering improved neuronal survival and maintaining synaptic integrity. Importantly, the activation of autophagy appears to be closely linked to redox-sensitive signaling pathways. Consequently, interventions that excessively suppress ROS generation may theoretically attenuate autophagy-related adaptive responses, further emphasizing the importance of maintaining physiological redox signaling in exercise-antioxidant interventions.

## 4 Exercise as a modulator of brain redox homeostasis

### 4.1 Types of exercise

Various modalities of physical activity yield divergent impacts on cognitive function and redox equilibrium. Aerobic exercise, encompassing activities such as running, cycling, and swimming, has been the subject of extensive investigation and is recognized for its ability to enhance cardiovascular health, augment oxygen supply to cerebral structures, and activate endogenous antioxidant defense mechanisms. Resistance training, which includes both weight lifting and bodyweight exercises, promotes muscular strength and has been correlated with diminished oxidative stress and inflammatory responses. High-intensity interval training (HIIT) integrates brief episodes of vigorous activity interspersed with recovery intervals and has been evidenced to elicit substantial mitochondrial adaptations and antioxidant responses. Mind–body interventions, including yoga and tai chi, have the capacity to alleviate stress and influence oxidative homeostasis via neuroendocrine pathways. In the context of NDDs such as AD and PD, customized exercise regimens may confer varying advantages contingent upon the stage of the disease and the individual’s functional capacity (48–50). Importantly, the neuroprotective effects of exercise are increasingly attributed to hormetic mechanisms, whereby transient increases in metabolic and oxidative stress stimulate adaptive antioxidant, mitochondrial, and neurotrophic responses. The magnitude of these adaptations may vary according to exercise modality, intensity, and duration, highlighting the importance of individualized exercise prescriptions in NDDs.

### 4.2 Molecular effects of exercise

**Figure 1:**
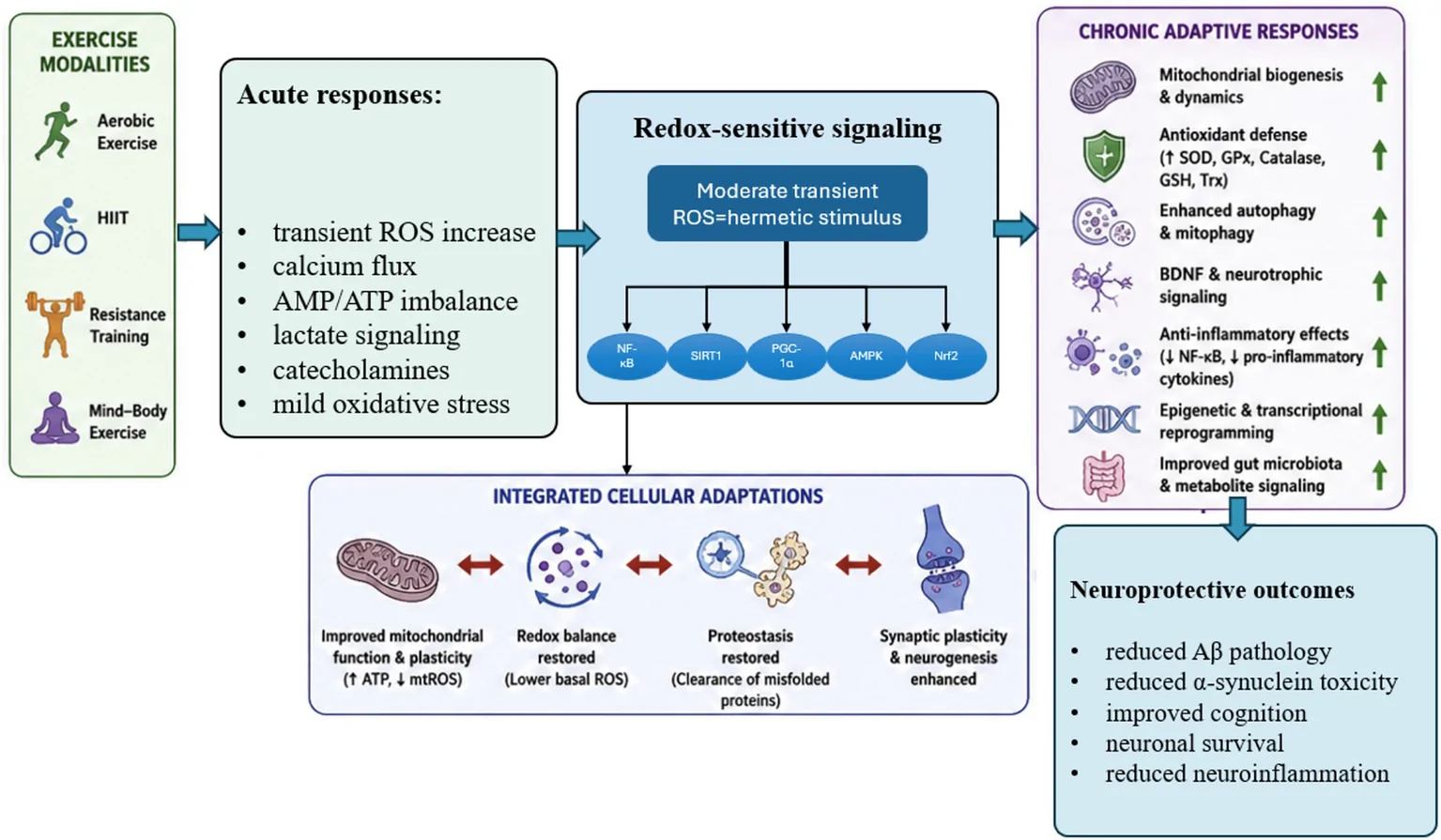
Exercise-induced redox hormesis and adaptive neuroprotection in neurodegenerative diseases.

At the molecular level, physical exercise significantly impacts a multitude of signaling pathways that are integral to redox regulation, mitochondrial functionality, and neuronal preservation (Figure 1). A principal mechanism underlying this influence is the activation of the Nrf2, which augments the expression of antioxidant and detoxifying enzymes. Additionally, exercise catalyzes the synthesis of BDNF, thereby facilitating neurogenesis, synaptic plasticity, and cognitive capabilities. Exercise enhances mitochondrial biogenesis via the activation of PGC-1α, which results in improved energy metabolism and diminished production of ROS. Notably, these adaptations are partially initiated by transient increases in ROS generated during exercise. Rather than acting solely as harmful molecules, physiological ROS function as signaling mediators that activate redox-sensitive pathways involved in mitochondrial biogenesis, antioxidant defense, autophagy, and neuroplasticity. It also modulates the process of autophagy, promoting the removal of damaged proteins and organelles, an aspect particularly pertinent in the context of NDDs. The anti-inflammatory effects, mediated through the attenuation of pro-inflammatory cytokines, further substantiate neuroprotective mechanisms. These molecular adaptations underscore the pivotal role of physical exercise in sustaining brain redox equilibrium and functional integrity (26, 51, 52). This concept provides a mechanistic basis for exercise-induced hormesis and may help explain why excessive antioxidant supplementation can, under certain conditions, attenuate adaptive exercise responses by suppressing ROS-dependent signaling pathways.

Different exercise modalities, including aerobic exercise, high-intensity interval training (HIIT), resistance training, and mind–body exercise, initiate acute physiological responses characterized by transient increases in reactive oxygen species (ROS), calcium flux, AMP/ATP imbalance, lactate signaling, catecholamine release, and mild oxidative stress. Rather than causing detrimental damage, these moderate and transient redox changes may act as hormetic stimuli that activate redox-sensitive signaling pathways, including NF-κB, SIRT1, PGC-1α, AMPK, and Nrf2. Repeated activation of these pathways may promote adaptive responses, including enhanced mitochondrial biogenesis and dynamics, upregulation of endogenous antioxidant defenses, improved autophagy and mitophagy, increased BDNF-mediated neurotrophic signaling, suppression of pro-inflammatory pathways, epigenetic remodeling, and modulation of gut microbiota-related metabolic signaling. These coordinated adaptations may contribute to integrated cellular remodeling characterized by improved mitochondrial plasticity, restoration of redox balance, enhanced proteostasis, and increased synaptic plasticity and neurogenesis. Collectively, these mechanisms have the potential to contribute to neuroprotective outcomes, including reduced amyloid-*β* (Aβ) pathology, attenuation of *α*-synuclein toxicity, decreased neuroinflammation, enhanced neuronal survival, and improved cognitive function. The figure provides a conceptual framework illustrating the transition from acute exercise-induced redox signaling to adaptive neuroprotective responses through exercise-induced hormesis. Importantly, the magnitude and extent of these adaptations are context-dependent and may vary according to exercise modality, intensity, duration, disease stage, individual redox status, and experimental or clinical conditions; therefore, the depicted pathways should not be interpreted as uniformly activated across all neurodegenerative disease settings.

## 5 Antioxidant systems: classification and biological roles

### 5.1 Vitamin antioxidants

Vitamin antioxidants represent a critical class of dietary compounds that play a significant role in safeguarding the brain from oxidative stress. Vitamin C, a hydrophilic antioxidant, effectively scavenges ROS within the cytosol and extracellular fluid, facilitates the regeneration of other antioxidants such as vitamin E, and plays a pivotal role in neurotransmitter biosynthesis. Vitamin E, an amphiphilic antioxidant, serves to protect cellular membranes from lipid peroxidation by neutralizing peroxyl radicals, thereby ensuring the preservation of membrane integrity. Vitamin A and its derivatives contribute to the antioxidant defense indirectly by modulating gene expression and promoting neuronal differentiation and plasticity. These vitamins exert influence over signaling pathways such as the Nrf2, thereby enhancing endogenous antioxidant responses. Insufficiencies in these vitamins have been linked to heightened oxidative damage and cognitive deterioration in conditions such as AD (53–55).

### 5.2 Mineral antioxidants

Minerals are of paramount importance as cofactors for antioxidant enzymes and in sustaining redox equilibrium within the central nervous system. Selenium serves as a vital constituent of glutathione peroxidases and thioredoxin reductases, which are essential for the detoxification of hydrogen peroxide and lipid hydroperoxides. Zinc enhances antioxidant defense mechanisms by stabilizing cellular membranes, inhibiting the activity of NADPH oxidase, and functioning as a structural element of superoxide dismutase (SOD). Copper and manganese are likewise critical to the various isoforms of SOD that facilitate the dismutation of superoxide radicals into less reactive entities. Iron, while indispensable for standard cellular operations, has the potential to catalyze the generation of highly reactive hydroxyl radicals through Fenton chemistry in states of dysregulation. Dysregulations in these minerals are frequently identified in NDDs such as PD, exacerbating oxidative stress and neuronal injury (56–58).

### 5.3 Endogenous antioxidants

Endogenous antioxidants are produced internally within the organism and serve critical functions in cellular protection against oxidative stress. Coenzyme Q10 (CoQ10) represents a fundamental element of the mitochondrial electron transport chain, acting as both an electron transporter and a powerful lipid-soluble antioxidant, thereby safeguarding membranes and mitochondrial constituents from oxidative harm. *α*-Lipoic acid is a multifaceted antioxidant capable of existing in both oxidized and reduced states, adept at scavenging ROS, regenerating other antioxidants such as vitamins C and E, and chelating metal ions. Notably, *α*-lipoic acid also augments mitochondrial functionality and facilitates energy metabolism. Both CoQ10 and α-lipoic acid have been demonstrated to influence redox-sensitive signaling pathways, including the Nrf2, and may confer neuroprotective advantages in pathological conditions such as AD and PD (59, 60).

### 5.4 Flavonoids

Flavonoids represent a heterogeneous assembly of polyphenolic compounds that are extensively prevalent in a variety of fruits, vegetables, and plant-derived beverages, demonstrating pronounced antioxidant and neuroprotective attributes. Specific compounds, including quercetin, epigallocatechin gallate (EGCG), luteolin, and apigenin, are capable of directly scavenging ROS, chelating metal ions, and inhibiting oxidative enzymes. In addition to their antioxidant capabilities, flavonoids significantly influence crucial signaling pathways, such as the Nrf2, thereby augmenting endogenous defense mechanisms. They exhibit anti-inflammatory properties, enhance mitochondrial functionality, and facilitate synaptic plasticity and cognitive performance. Notably, numerous flavonoids possess the ability to traverse the blood–brain barrier, enabling them to exert direct influences on neuronal cells. These characteristics render flavonoids as promising therapeutic agents for alleviating oxidative stress and decelerating the advancement of NDDs (61, 62).

### 5.5 Antioxidants and redox hormesis

Although antioxidants play important roles in limiting oxidative damage and supporting neuronal function, growing evidence indicates that their biological effects are context-dependent. ROS not only contribute to cellular injury but also function as signaling molecules involved in adaptive responses such as Nrf2 activation, mitochondrial biogenesis, autophagy, and neuroplasticity. Consequently, excessive antioxidant supplementation may attenuate these beneficial redox-sensitive pathways, a phenomenon commonly referred to as the “antioxidant paradox.” This concept suggests that the neuroprotective efficacy of antioxidant interventions depends not only on antioxidant capacity but also on the preservation of physiological redox signaling. Therefore, factors such as antioxidant type, dosage, bioavailability, and timing of administration should be carefully considered when evaluating their therapeutic potential in NDDs (32–35).

## 6 The effects of antioxidants and exercise in experimental models

### 6.1 AD models

#### 6.1.1 Effect of exercise alone

Preclinical studies consistently show that aerobic exercise and HIIT improve cognitive performance, memory, and spatial learning in experimental models of AD. These benefits are commonly associated with activation of Nrf2 signaling, increased SOD and catalase (CAT) activity, reduced Aβ accumulation, and enhanced hippocampal neurogenesis. Improvements in behavioral outcomes, including performance in the Morris water maze and novel object recognition tests, further support the role of exercise as an effective non-pharmacological strategy for attenuating cognitive decline in AD.

Recent investigations further indicate that the neuroprotective attributes of physical exercise may, in part, involve the modulation of gut microbiota alongside the pathways of gut-brain signaling. Exercise-induced modifications in microbial diversity and metabolites derived from microbiota, such as short-chain fatty acids (SCFAs), have been linked to diminished neuroinflammation, enhanced mitochondrial functionality, and improved neuronal plasticity in experimental models of AD. Antioxidant interventions may play a role in maintaining the integrity of the intestinal barrier and promoting immune homeostasis, thereby potentially affecting redox balance and cognitive performance through mechanisms associated with microbiota (63, 64). Collectively, findings from experimental AD models indicate that the neuroprotective effects of exercise extend beyond improvements in behavioral performance and involve coordinated modulation of redox homeostasis, neurotrophic signaling, mitochondrial function, and gut–brain communication. Across different exercise modalities, activation of the Nrf2–BDNF axis, enhancement of endogenous antioxidant defenses, and preservation of mitochondrial plasticity emerge as recurring mechanistic patterns. However, substantial heterogeneity exists across experimental studies. Variability in exercise modality, intensity, and duration, together with differences in antioxidant type, dosage, bioavailability, disease stage, genetic background, age, sex, and outcome measures, likely contributes to inconsistencies in the magnitude and nature of reported benefits.

**Figure 2:**
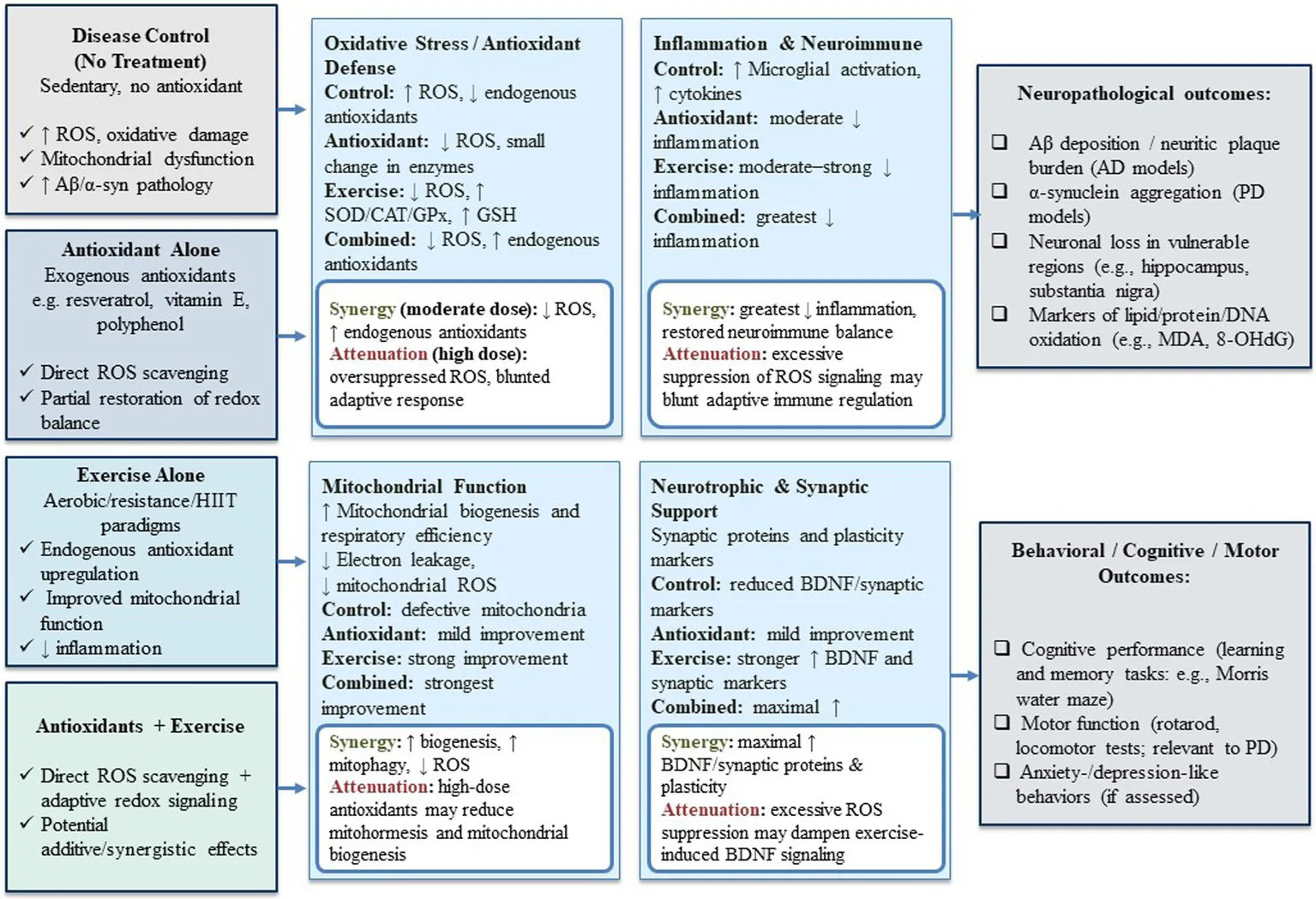
Context-dependent effects of exercise and antioxidant supplementation on redox signaling and neuroprotection in neurodegenerative diseases.

An investigation evaluated alterations in neuroprotective proteins—proBDNF, BDNF, and humanin—in plasma neuronal-derived extracellular vesicles (NDEVs) from 95 individuals with mild to moderate AD enrolled in the ADEX randomized controlled trial. Participants were assigned to exercise or control groups, with samples collected at baseline and after 16 weeks. Exercise induced a significant increase in NDEV-associated proBDNF, BDNF, and humanin, with a more pronounced effect in APOE ε4 carriers, whereas the control group remained unchanged. Notably, baseline inter-biomarker correlations were largely preserved after the intervention, suggesting stability of systemic neurotrophic relationships despite intervention-induced modulation. Conversely, exerkine-associated NDEVs did not show measurable changes, indicating a selective rather than global vesicular response to exercise (65) (Table 1 and Figure 2). Stein et al. investigated whether aerobic exercise modulates circulating neurotrophins and cognitive performance in AD patients. A total of 34 subjects were allocated to a control group (*n* = 16) and an intervention group (n = 18), with the latter performing moderate treadmill exercise three times per week for 12 weeks. Outcomes included plasma BDNF, vascular endothelial growth factor 165, and insulin-like growth factor 1 as biological markers, alongside cognitive performance and aerobic capacity. Following intervention, participants demonstrated improved aerobic fitness and preservation of executive function. However, circulating neurotrophin levels remained unchanged despite functional and fitness improvements (66). Notably, the effects of exercise on circulating neurotrophic biomarkers are not entirely consistent across studies. While some investigations report significant increases in BDNF-related signaling, others demonstrate functional and cognitive improvements without measurable changes in circulating neurotrophin concentrations. These discrepancies may reflect differences in disease severity, intervention characteristics, biomarker sources, and assessment methods. They also suggest that exercise-induced neuroprotection is likely mediated through multiple complementary pathways rather than a single molecular marker.

**Table 1:** Summary of reported findings on exercise-related changes in biomarkers, neurogenesis, and cognition in Alzheimer’s disease.

Population/model	Intervention	Duration	Mechanism	Findings	Evidence strength	Limitations/variability	Study
95 patients with mild–moderate Alzheimer’s disease	Exercise vs. control (RCT)	16 weeks	Exercise-induced modulation of neuroprotective signaling in brain-derived extracellular vesicles (↑ proBDNF, BDNF, humanin; APOE ε4-dependent response)	↑ proBDNF, BDNF, humanin in exercise group (especially APOE ε4 carriers); no change in controls; exerkines unchanged	High (RCT, relatively large sample, mechanistic biomarker analysis)	Biomarker-based (not direct clinical endpoints); moderate duration; heterogeneity due to APOE genotype; unclear dose–response (intensity/type not fully dissected)	(65)
34 AD patients (CG = 16, TG = 18)	Moderate aerobic treadmill training	12 weeks(3×/week)	Aerobic exercise–induced neurotrophin regulation (BDNF, VEGF165, IGF-1) and cognitive support pathways	No significant changes in neurotrophins; maintenance of executive function; ↑ aerobic fitness	Moderate (controlled human study but small sample and negative biomarker findings)	Small sample size; short intervention; no biomarker response despite physiological adaptation; possible insufficient intensity/duration for neurotrophic changes	(66)
3xTg AD mouse model (C57Bl/6 background)	Running ± fluoxetine	11 months	Activity-induced neurogenesis (hippocampal neuroplasticity; BDNF-related pathways); pharmacological modulation comparison	↑ survival of newborn neurons with running; no change in Aβ, tau, or BDNF; fluoxetine ineffective	Moderate–Low (strong mechanistic animal study but translational limitations and model variability)	Very long duration but animal model; lack of behavioral phenotype in this strain (model limitation); sex-specific sample (female only); conflicting drug–exercise interaction; limited translational validity	(67)
Aβ1-42-induced AD rat model	Treadmill (fed vs. fasted ± IF)	4 weeks (5 days/week)	β-hydroxybutyrate (βHB)-mediated activation of PKA/CREB/BDNF signaling; metabolic–neurotrophic coupling	↑ BDNF/PKA/CREB; ↑ βHB; improved cognition; improved cognition and BDNF/PKA/CREB signaling across intervention groups, with the largest effects observed in the fasted-exercise condition	High (well-controlled animal factorial design, mechanistic pathway analysis)	Animal model limitation; short duration; Aβ injection model does not fully replicate AD pathology; limited translational validity; no long-term follow-up	(68)
d-galactose + AlCl₃ AD rat model	Treadmill exercise	10 weeks	Activation of AMPK/SIRT1/PGC-1*α* → mitochondrial biogenesis; FNDC5/BDNF axis; MG53-mediated cellular repair	↑ MG53; ↑ mitochondrial biogenesis; ↓ plasma tau; improved behavior	Moderate–High (robust mechanistic animal study with multi-tissue molecular validation)	Induced chemical AD model; limited human relevance; unclear sample size detail; high mechanistic complexity but no translational confirmation	(69)
23 elderly (mild–moderate AD)	Multimodal training	12 weeks	Neuroplasticity and anti-inflammatory modulation (↑ BDNF, ↓ IL-2); motor-cognitive integration effects	↑ BDNF; ↓ IL-2; improved cognition & mobility	Moderate (human study but non-randomized, small sample size)	Non-randomized design; small intervention group (n = 7); mixed control structure; potential selection bias; short duration; limited biomarker panel	(70)
AD rat model	Low/mod/high intensity exercise	4 weeks (5 days/week, 20 min/day)	Intensity-dependent regulation of neurotrophic factors (↑ BDNF most in high-intensity group)	All exercise intensities improved outcomes, although neurotrophic responses were greatest following high-intensity exercise	High (controlled animal dose–response design with intensity stratification)	Animal model limitation; short duration; lack of translational human data; intensity definitions may not directly map to clinical exercise prescription	(71)
Aβ1-42-induced AD rats	Aerobic rehabilitation	4 weeks (5 days/week)	BDNF and TGF-β1 signaling pathways in hippocampus; neuroplasticity and synaptic repair mechanisms	↑ BDNF and TGF-β1; improved memory	Moderate–High (controlled animal study with molecular + behavioral outcomes)	Small sample size; single species/sex; invasive AD induction model; limited clinical translation	(72)
Aβ1-42 AD rat model	High-intensity interval training	8 weeks (after 2-week adaptation)	BDNF/TrkB activation, oxidative stress reduction (↑ SOD, CAT; ↓ lipid peroxidation), reduction of amyloid pathology	↑ BDNF/TrkB; enhanced antioxidant defenses and reduced oxidative damage; ↓ neuritic plaques	High (robust mechanistic animal study with behavioral, biochemical, and histological endpoints)	Animal model only; HIIT protocol highly controlled and may not reflect human adherence; surgical Aβ model limitations; no long-term follow-up	(73)

AD, Alzheimer’s disease; NDEVs, neuron-derived extracellular vesicles; BDNF, brain-derived neurotrophic factor; proBDNF, precursor brain-derived neurotrophic factor; CTSB, cathepsin B; NGF, nerve growth factor; TrkB, tropomyosin receptor kinase B; Aβ, amyloid-beta; APOE, apolipoprotein E; HIIT, high-intensity interval training; PKA, protein kinase A; CREB, cAMP response element-binding protein; βHB, beta-hydroxybutyrate; AMPK, AMP-activated protein kinase; SIRT1, sirtuin 1; PGC-1α, peroxisome proliferator-activated receptor gamma coactivator 1-alpha; MG53, mitsugumin 53; IL-2, interleukin-2; TGF-β1, transforming growth factor beta 1; CAT, catalase; SOD, superoxide dismutase.

Note: The table provides a structured summary of evidence across preclinical and clinical studies. Findings are presented in a balanced manner and include both positive and neutral/absent effects were reported. Exercise-induced adaptations may vary depending on intervention type, intensity, duration, and participant characteristics, reflecting substantial inter-study heterogeneity. In addition, preclinical studies generally demonstrate stronger mechanistic responses compared to human trials, highlighting an important translational gap. Gut microbiota-related biomarkers were not consistently assessed across the included studies and are therefore not included in the current table.

Overall, exercise-induced neuroprotective responses varied according to exercise modality, intensity, intervention duration, disease stage, and experimental model characteristics, indicating substantial context dependency across studies. Findings should be interpreted in light of differences in exercise protocols, disease models, intervention duration, and biomarker assessment methods, which contribute to context-dependent variability across studies.

Marlatt et al. investigated the effects of long-term physical exercise and pharmacological intervention on neurogenesis and AD pathology in a 3xTg mouse model backcrossed to C57BL/6 background. Mice received fluoxetine, voluntary running, or combined interventions for 11 months. Despite substantial intraneuronal Aβ accumulation in cortex and amygdala, spatial memory deficits were absent, suggesting a dissociation between amyloid burden and cognitive impairment in this model. Exercise did not alter amyloid or tau pathology nor BDNF levels, indicating limited impact on core pathological markers. However, it selectively enhanced survival of newly generated neurons during mid-life, suggesting a pro-neurogenic but not disease-modifying effect. Fluoxetine showed no beneficial effects and, when combined with exercise, attenuated voluntary running (67). Importantly, the absence of substantial effects on amyloid burden, tau pathology, or BDNF signaling in certain models does not necessarily negate the neuroprotective potential of exercise. Instead, these findings suggest that exercise may preferentially enhance neuronal resilience, neurogenesis, and functional adaptation rather than directly modifying all pathological hallmarks of AD. Such observations underscore the complexity of translating molecular changes into disease-modifying outcomes and highlight an important source of heterogeneity across experimental studies. Kirkbir et al. examined moderate treadmill exercise under fasted and fed conditions in an Aβ₁_−_₄₂-induced AD rat model. Aged Wistar rats were divided into AD, intermittent fasting, exercise, combined intermittent fasting +exercise, and control groups. A*β* administration induced impairments in learning, memory, and hippocampal protein kinase A/CREB/BDNF signaling. Both fasting and exercise increased β-hydroxybutyrate levels, with the greatest elevation observed in the combined intervention group. All intervention groups showed cognitive and signaling improvements, while suggesting potentially complementary metabolic and neurotrophic adaptations (68).

Belviranlı et al. examined chronic treadmill exercise in a d-galactose and AlCl₃-induced sporadic AD rat model. Subjects were assigned to control, AD, exercise, or AD+exercise groups for 10 weeks. The AD phenotype was characterized by cognitive and motor impairments accompanied by elevated plasma tau levels and suppression of AMP-activated protein kinase (AMPK), Sirtuin 1, and peroxisome proliferator-activated receptor gamma coactivator 1-alpha (PGC-1α) signaling, together with reduced MG53 expression across brain and peripheral tissues, indicating impaired mitochondrial biogenesis and cellular repair mechanisms. Exercise intervention ameliorated behavioral deficits and restored plasma tau toward baseline, while upregulating the AMPK/PGC-1α/fibronectin type III domain-containing protein 5 (FNDC5)/BDNF axis, suggesting coordinated central–peripheral enhancement of mitochondrial and neurotrophic signaling. Remarkably, exercise significantly elevated MG53 expression at both gene and protein levels across cerebral, muscular, and circulatory systems, thereby uncovering an innovative neuroprotective mechanism that connects systemic cellular repair processes to the enhancement of cognitive resilience in the context of AD (69). Lopes et al. assessed a 12-week multimodal exercise program in elderly patients with mild to moderate AD. Participants (62–85 years) underwent a multimodal exercise program (aerobic, resistance, flexibility, and coordination training) for 12 weeks, compared with a control group receiving usual care. Exercise improved executive and attentional functions alongside gait, balance, and muscular strength. Additionally, increased plasma BDNF and reduced IL-2 levels, indicating simultaneous enhancement of neurotrophic support and anti-inflammatory regulation (70).

Lee et al. evaluated the effects of aerobic exercise at different intensities on cognitive and motor performance and neurotrophic signaling in an AD rat model. Thirty-two rats were assigned to control and low-, moderate-, and high-intensity exercise groups, with training performed for 20 min/day, 5 days/week over 4 weeks. All exercise intensities improved cognitive and motor outcomes as well as neurotrophic signaling compared with controls, indicating a general beneficial effect of aerobic activity. Notably, high-intensity exercise induced the greatest upregulation of BDNF and the strongest behavioral performance, suggesting a dose–response relationship between exercise intensity and neurotrophic activation (71). Fakhraei et al. investigated a 4-week aerobic rehabilitation exercise program in an Aβ₁_−_₄₂-induced AD rat model. Rats were allocated to Aβ-only, Aβ + exercise, and control groups. Exercise significantly increased hippocampal expression of BDNF and transforming growth factor beta 1 signaling components, accompanied by improved spatial memory performance, indicating coordinated activation of neurotrophic and neurorepair pathways in response to aerobic rehabilitation (72). An investigation evaluated short-duration HIIT with incremental overload in an Aβ₁_−_₄₂-induced AD rodent model. Forty male Wistar rats were assigned to Sham, Sham-trained, AD-untrained, and AD-trained groups and underwent an 8-week HIIT protocol following stereotactic hippocampal Aβ injection. HIIT training improved locomotor activity, learning, and memory performance in AD animals compared with untrained counterparts. These functional improvements were accompanied by enhanced antioxidant defenses (CAT, SOD), reduced lipid peroxidation, activation of BDNF/tropomyosin receptor kinase B (TrkB) signaling, and attenuation of neuritic plaque burden, indicating both redox and neurotrophic remodeling. These findings suggest that HIIT may exert multimodal neuroprotective effects through coordinated modulation of redox homeostasis, neurotrophic signaling, and amyloid pathology. The MRT was used to individualize training intensity and ensure controlled overload during the intervention (73).

Taken together, evidence from experimental AD models supports the concept that exercise promotes neuroprotection through interconnected effects on redox regulation, neurotrophic signaling, mitochondrial adaptation, and cellular quality-control mechanisms. A recurring observation across studies is that improvements in cognition and neuronal function are not always accompanied by parallel changes in every pathological marker, emphasizing the importance of mechanistic and functional outcomes in addition to classical disease hallmarks. From a hormetic perspective, moderate increases in ROS generated during exercise may serve as essential signaling molecules that activate adaptive pathways such as Nrf2, BDNF, autophagy, and mitochondrial biogenesis. Consequently, although antioxidant interventions are often beneficial, excessive suppression of ROS signaling could attenuate exercise-induced adaptive responses. The magnitude of this effect appears to be strongly influenced by antioxidant dosage and timing of administration. Low-to-moderate antioxidant doses may support redox homeostasis under conditions of excessive oxidative stress, whereas high-dose supplementation may excessively scavenge ROS and blunt exercise-induced activation of Nrf2, mitochondrial biogenesis, and mitohormetic signaling. Likewise, antioxidant administration immediately before or after exercise may interfere with ROS-dependent adaptive pathways to a greater extent than individualized or delayed supplementation strategies. This mechanism may partially explain the neutral, inconsistent, or attenuated effects reported in some studies and highlights the importance of antioxidant dosage, timing, disease stage, and intervention context when translating combined exercise–antioxidant strategies into clinical practice.

The figure summarizes the potential mechanistic effects of exercise, antioxidant supplementation, and their combination on oxidative stress, inflammation, mitochondrial function, and neurotrophic signaling. Exercise promotes adaptive redox signaling and hormetic responses through pathways including AMPK–SIRT1–PGC-1α and Nrf2, leading to enhanced mitochondrial biogenesis, endogenous antioxidant defense, and neuroplasticity. Antioxidant supplementation primarily limits excessive oxidative stress through direct and indirect modulation of ROS-dependent pathways. Under specific conditions, particularly when antioxidant dosing is moderate and physiological ROS signaling is preserved, combined interventions may exert additive or synergistic neuroprotective effects. However, excessive antioxidant supplementation may suppress adaptive exercise-induced redox signaling and attenuate mitohormesis, mitochondrial adaptation, and neurotrophic responses, illustrating the “antioxidant paradox.” Consequently, interactions between exercise and antioxidant supplementation should be considered context-dependent rather than uniformly synergistic, with outcomes varying according to antioxidant type and dose, timing of supplementation, exercise modality and intensity, disease stage, and baseline redox status. The figure presents a conceptual model and does not imply that combined interventions consistently produce superior outcomes compared with exercise or antioxidant monotherapy.

#### 6.1.2 Effect of antioxidant supplementation alone

Antioxidant supplementation in AD models demonstrates highly heterogeneous outcomes, suggesting that its efficacy is strongly dependent on molecular context, disease stage, genetic background, and target pathway specificity. Overall, antioxidant interventions primarily exert their effects through modulation of redox homeostasis, mitochondrial function, protein aggregation pathways, and neuroinflammatory signaling, rather than uniformly improving cognitive outcomes. A major mechanistic theme across studies is redox restoration and lipid peroxidation control, particularly through classical antioxidants such as vitamins C and E. Combined vitamin C/E supplementation has been shown to enhance systemic and central antioxidant status by increasing antioxidant levels in plasma and cerebrospinal fluid and reducing lipoprotein oxidizability. However, vitamin E alone increases circulating levels without significantly improving oxidative susceptibility, indicating that biochemical elevation does not necessarily translate into functional redox protection (74) (Table 2). Similarly, long-term supplementation with vitamins C and E leads to sustained biochemical improvements in cerebrospinal antioxidant capacity, yet fails to produce measurable cognitive benefits, highlighting a dissociation between redox normalization and clinical outcomes (75). Importantly, these findings illustrate a recurring pattern in antioxidant research, whereby improvements in biochemical markers of oxidative stress do not consistently translate into functional or cognitive benefits. This discrepancy suggests that modulation of oxidative status alone may be insufficient to alter disease progression and highlights the need to consider broader mechanisms, including neuroplasticity, mitochondrial adaptation, and cellular stress responses. A second mechanistic axis involves metal homeostasis and genotype-dependent oxidative vulnerability. Zinc supplementation, for example, exhibits divergent effects depending on ApoE genotype, where ApoE ε4 carriers demonstrate worsened spatial memory following zinc administration. This cognitive decline correlates with altered Aβ dynamics and zinc transporter expression, indicating that dysregulated metal–amyloid interactions may exacerbate oxidative and synaptic dysfunction rather than confer protection (76). The genotype-dependent effects observed with zinc further emphasize that antioxidant interventions are not universally beneficial and that therapeutic responses may depend on disease biology, genetic susceptibility, and baseline redox status. Such observations represent important sources of heterogeneity across experimental and clinical studies, together with differences in antioxidant class, dosage, bioavailability, treatment duration, disease stage, and genetic background.

**Table 2:** Overview of antioxidant interventions in Alzheimer’s disease: Findings from preclinical and clinical studies.

Population/model	Intervention	Dose	Duration	Mechanism	Findings	Evidence strength	Limitations/variability	Study
AD patients (n = 10 per group)	Vitamin E + Vitamin C or Vitamin E alone	400 IU vitamin E and 1,000 mg vitamin C	1 month	Increased antioxidant capacity in plasma/CSF → reduced lipoprotein oxidation susceptibility	E + C increased plasma and CSF antioxidant levels and reduced lipoprotein oxidation, whereas vitamin E alone increased vitamin concentrations without significantly improving oxidative susceptibility.	Moderate (human biochemical intervention but very small sample size, short-term mechanistic endpoint)	Very small sample (n = 10/group); short duration; no clinical cognitive outcomes; biochemical endpoints only; limited generalizability	(74)
AD patients (n = 23; 12 supplemented, 11 controls)	Vitamin E + Vitamin C	400 IU vitamin E and 1,000 mg vitamin C	1 year	CSF antioxidant enrichment → reduced lipid peroxidation (*in vitro* CSF oxidation assay)	↑ CSF antioxidant levels and ↓ lipid oxidation, but no measurable cognitive improvement	Moderate (longitudinal human study but non-randomized/open-label design)	Non-randomized design; small sample; open-label bias; no significant clinical benefit; incomplete CSF sampling (subset only)	(75)
Mouse late-onset AD (CRND8/CRND8-E4)	Dietary zinc	10 ppm Zn (ZnCO3)	4 months	Metal–amyloid interaction: Zn enhances Aβ aggregation via ApoE ε4-dependent binding; synaptic dysfunction	CRND8/E4: ↓ spatial memory, ↑ soluble & insoluble Aβ	Moderate–High (controlled genetic animal model with mechanistic gene–environment interaction analysis)	Animal model (late-onset AD approximation but not fully human-representative); dietary dose translation unclear; strong genotype dependence (ApoE ε4 variability); no therapeutic intervention—risk factor model	(76)
Aged Aβ-induced AD rats	Coenzyme Q10	50 mg/kg	4 weeks	Mitochondrial antioxidant action → ↓ oxidative stress (↓MDA, ↓TOS), ↑ antioxidant capacity (↑TAC, ↑TTG), improved synaptic plasticity (LTP)	↑ learning & memory, ↑ synaptic plasticity, ↓ MDA & TOS, ↑ TAC & TTG	High (controlled animal study with behavioral + biochemical + electrophysiological endpoints)	Animal model limitation; pre-treatment design (not therapeutic reversal only); short duration; limited translational relevance to chronic AD	(77)
P301S Tau transgenic mice	α-Lipoic acid	α-lipoic acid 3 mg/kg/day or 10 mg/kg/day (i.p., daily with 1-day break every 3 days)	10 weeks	Anti-tauopathy effects: ↓ Tau hyperphosphorylation; ↓ calpain1 activity; ↓ calcium dysregulation; inhibition of ferroptosis (↓ iron overload, lipid peroxidation, inflammation)	↓ Tau hyperphosphorylation, ↓ apoptosis, ↓ ferroptosis, ↓ inflammation, ↑ cognitive function	High (strong mechanistic animal model of tauopathy with multi-pathway validation)	Dose and exact duration not clearly stated; animal-only model; translational uncertainty; complex multi-target effects complicate causality attribution	(78)
Wistar rats with chronic stress + scopolamine	Quercetin	25 mg/kg/day	1 month (with last 9 days SCOP induction)	Anti-inflammatory and neuroprotective effects; modulation of cytokines (IL-6, TNF-α); effects on APP/APLP2 expression; shift toward non-amyloidogenic pathways	↑ cognitive function, ↓ anxiety, ↓ IL-6 & TNF-α, ↓ hippocampal Aβ	Moderate (animal behavioral + molecular study, but complex stress/confounding model)	Mixed disease induction model (stress + scopolamine); gene expression paradox (APP increase in some conditions); unclear translation to AD pathology; missing exact AD-specific model validity	(79)
APPsw transgenic mice	EGCG	20 mg/kg i.p. or 50 mg/kg oral	6 months	Promotes non-amyloidogenic APP processing (↑ α-secretase), reduces Aβ production, modulates tau phosphorylation	Reduced amyloid and tau pathology with improvements in cognition; efficacy varied according to dose and route of administration	High (long-term *in vivo* transgenic model with behavioral + histological + biochemical endpoints)	Animal model limitation; high-dose long-term supplementation not directly translatable; variability in route-dependent efficacy (i.p. > oral for cognition)	(80)
APPsw transgenic mice & neuron-like cells	EGCG (*in vitro* & *in vivo*)	EGCG 20 mg/kg/day (i.p.); ICV 10 μg/mouse (single dose)	60 days	Enhances α-secretase activity (TACE/ADAM17), shifts APP processing to non-amyloidogenic pathway, reduces Aβ generation	↓ Aβ levels & plaques, ↑ sAPPα	High (multi-level mechanistic study: cell + animal validation)	Dose and exposure duration not fully standardized across experiments; translational uncertainty; reliance on transgenic overexpression models	(81)
5XFAD x Gulo KO mice	High-dose vitamin C	0.66 g/L (early phase) → 3.3 g/L (after 8 weeks) in drinking water	6 months	Antioxidant and mitochondrial protection; improvement of BBB integrity; modulation of mitochondrial morphology and oxidative stress	↓ amyloid plaques, ↓ BBB disruption, ↓ mitochondrial alterations	High (genetically modified AD model with mechanistic relevance to human vitamin C dependency)	Exact duration not clearly reported; extreme genetic manipulation model (Gulo KO) reduces direct human comparability; dietary dosing not directly translatable; lack of behavioral detail in excerpt	(82)

AD, Alzheimer’s disease; CSF, cerebrospinal fluid; ApoE, apolipoprotein E; Aβ, amyloid-beta; APP, amyloid precursor protein; Q10, coenzyme Q10; LA, α-lipoic acid; QUER, quercetin; EGCG, epigallocatechin-3-gallate; BBB, blood–brain barrier; 5XFAD, five familial Alzheimer’s disease mutations; Gulo KO, ι-gulono-γ-lactone oxidase knockout.

Note: The studies included in this table show heterogeneous findings regarding antioxidant supplementation and neuroprotective outcomes. While several preclinical studies report beneficial effects on oxidative stress, neuroplasticity, and AD-related pathology, clinical trials demonstrate inconsistent or modest effects. This discrepancy may reflect a translational gap between animal models and human studies, potentially influenced by differences in bioavailability, blood–brain barrier permeability, and study design. Additionally, emerging evidence suggests that high-dose antioxidant supplementation may not always enhance, and may potentially attenuate, exercise-induced adaptive responses (“antioxidant paradox”). Gut microbiota-related biomarkers were not assessed in the included studies, despite their emerging relevance in the gut–brain axis and neurodegeneration.

Overall, antioxidant interventions demonstrated substantial heterogeneity across studies. Their efficacy depended on antioxidant class, dosage, route of administration, disease stage, genetic background, baseline redox status, and experimental model characteristics. Biochemical improvements did not consistently translate into cognitive or functional benefits.

Similarly, *α*-lipoic acid exerts broader pleiotropic effects by targeting multiple degenerative pathways simultaneously. Beyond reducing oxidative stress and iron accumulation, *α*-lipoic acid modulates calcium dysregulation, suppresses apoptosis, and attenuates ferroptosis-related neurotoxicity, while also improving tau pathology and cognitive outcomes, indicating integrated regulation of redox imbalance and protein aggregation pathways (77). Beyond reducing oxidative stress and iron accumulation, α-lipoic acid modulates calcium dysregulation, suppresses apoptosis, and attenuates ferroptosis-related neurotoxicity, while also improving tau pathology and cognitive outcomes, indicating integrated regulation of redox imbalance and protein aggregation pathways (78). Polyphenolic compounds further expand these mechanisms through neuroinflammation and amyloid processing modulation. Quercetin reduces pro-inflammatory cytokines, modulates APP/APLP2 expression, and shifts amyloid precursor protein processing toward non-amyloidogenic pathways, thereby reducing amyloid burden and improving behavioral outcomes across stress and non-stress conditions (79). Likewise, EGCG from green tea exerts dual anti-amyloid and anti-tau effects by enhancing *α*-secretase activity, reducing Aβ accumulation, and improving cognitive performance, with both *in vivo* and *in vitro* evidence supporting its role in promoting non-amyloidogenic APP processing (80, 81). Finally, vitamin C supplementation in genetic models of impaired endogenous antioxidant synthesis demonstrates dose-dependent neurovascular and mitochondrial protection, including reduced amyloid deposition, improved blood–brain barrier integrity, and enhanced mitochondrial function, suggesting that antioxidant efficacy may depend critically on physiological baseline status and dosage thresholds (82).

Taken together, the most consistent benefits of antioxidant supplementation appear to arise when interventions influence multiple interconnected pathways rather than solely reducing oxidative stress. Compounds that simultaneously modulate mitochondrial function, neuroinflammation, protein aggregation, and synaptic plasticity generally demonstrate broader neuroprotective effects than antioxidants acting primarily as direct free-radical scavengers. This observation has important translational implications, suggesting that pathway-specific targeting may be more effective than generalized antioxidant replacement strategies. Collectively, these findings indicate that antioxidant supplementation alone does not act through a single unified pathway but rather through multiple partially overlapping mechanisms. However, its therapeutic efficacy is highly context-dependent, and in many cases biochemical improvements in oxidative markers do not translate into consistent cognitive benefits. From the perspective of hormesis and the antioxidant paradox, these observations also suggest that excessive suppression of ROS may not always be advantageous. Low-to-moderate antioxidant supplementation may help restore redox homeostasis under conditions of excessive oxidative stress, whereas high-dose supplementation could excessively scavenge ROS required for adaptive signaling. Furthermore, the timing of antioxidant administration may influence outcomes, as prolonged or inappropriately timed supplementation could interfere with endogenous stress-response pathways involved in mitochondrial adaptation and neuronal resilience. Although most studies in this section evaluated antioxidant supplementation in isolation rather than in combination with exercise, adaptive redox signaling is increasingly recognized as an important regulator of mitochondrial function, endogenous antioxidant defenses, and neuronal resilience. Consequently, antioxidant dosage, timing of administration, treatment duration, and baseline oxidative status may critically influence whether antioxidant interventions support or inadvertently attenuate beneficial stress-response pathways. Therefore, antioxidant monotherapy is unlikely to provide uniform neuroprotection across all disease contexts, reinforcing the need for precision-based approaches that consider disease stage, genetic background, redox status, and potential interactions with exercise-induced adaptive signaling.

#### 6.1.3 Effect of combined exercise and antioxidants

Combined exercise and antioxidant interventions frequently demonstrate broader or more robust neuroprotective effects than monotherapies; however, these effects are highly context-dependent, and their magnitude and direction vary according to the antioxidant used, exercise modality, and molecular pathways targeted. Across studies, three major mechanistic patterns emerge: (i) convergence on redox homeostasis and mitochondrial integrity, (ii) modulation of neurotrophic and metabolic signaling pathways, and (iii) regulation of inflammation, autophagy, and protein aggregation processes. A dominant mechanistic theme involves enhancement of redox regulation and mitochondrial resilience. Combined interventions such as HIIT with vitamin E or aerobic exercise with vitamin C consistently activate antioxidant defense systems, including NRF2 signaling, SOD, CAT, and glutathione-related pathways, while simultaneously reducing oxidative damage markers such as MDA and Aβ accumulation. These interventions also suppress pro-inflammatory signaling such as nuclear factor kappa B (NF-κB) and apoptotic cascades, indicating integrated regulation of oxidative stress and cell survival pathways (83–85) (Table 3). Collectively, these findings suggest coordinated activation of redox-sensitive transcriptional programs alongside suppression of oxidative injury. Importantly, the benefits of combined interventions do not appear to result solely from maximal suppression of oxidative stress. Rather, many of the observed effects are consistent with the concept of adaptive redox signaling, whereby moderate ROS production functions as a physiological trigger for Nrf2 activation, mitochondrial remodeling, and neurotrophic responses. This observation suggests that preservation of redox signaling may be as important as the reduction of oxidative damage itself. A second convergent mechanism is metabolic and neurotrophic pathway modulation, particularly involving CoQ10, *α*-lipoic acid, and exercise-based interventions. Combined HIIT and CoQ10 improve cognitive performance and restore antioxidant capacity (thiols, CAT, glutathione peroxidase), while reducing neuronal degeneration and lipid peroxidation, indicating structural and functional neuroprotection (86). In parallel, activation of the FNDC5/irisin/BDNF axis suggests that metabolic signaling is tightly linked to synaptic plasticity and amyloid modulation (87). Similarly, exercise combined with *α*-lipoic acid enhances glucose transporter expression and BDNF signaling while strengthening antioxidant enzyme activity, although certain apoptotic and stress-response pathways remain unaffected, indicating selective rather than global interactions between exercise and antioxidant supplementation (88). Taken together, these findings suggest that the most consistent benefits of combined interventions arise when exercise and antioxidant compounds converge on complementary metabolic and neurotrophic pathways rather than simply amplifying antioxidant capacity. Mineral-related interventions also contribute to multimodal neuroprotection through combined modulation of oxidative stress and signaling pathways. Melatonin and zinc sulfate in conjunction with physical and mental activities attenuate AlCl3-induced neurodegeneration, improve cognitive performance, and reduce AD-related biomarkers, including Aβ, and tau. These effects are associated with restoration of glycogen synthase kinase 3 beta, wingless/integrated signaling pathway, and beta-catenin signaling and suppression of systemic oxidative stress and inflammation, indicating integrated neurovascular and metabolic regulation (89). A third major theme involves multimodal regulation of neuroinflammation, autophagy, and protein aggregation, particularly with polyphenols and flavonoids. Quercetin combined with physical activity reduces oxidative stress and improves cognitive outcomes in streptozotocin-induced models through additive enhancement of antioxidant defenses (90) (Table 4). EGCG-based interventions show partial overlap with exercise effects, improving behavioral performance and reducing Aβ burden, although synergy is not consistently additive across all parameters (91). More integrated effects are observed with luteolin, where combined exercise promotes autophagy-dependent cognitive improvement, metabolic pathway reprogramming, and suppression of neuroinflammation, indicating that autophagy acts as a key mechanistic mediator of neuroprotection (92, 93). Similarly, other flavonoids such as apigenin and epicatechin exhibit coordinated effects on cholinergic function, oxidative stress, inflammatory signaling, and neurotrophic pathways. For example, epicatechin combined with exercise activates Akt/GSK-3/CREB signaling and increases BDNF expression, suggesting convergence on survival and synaptic plasticity pathways (94, 95). Collectively, flavonoid-based interventions appear to exert broader biological effects than direct antioxidant activity alone, integrating redox regulation with autophagy, inflammatory control, and synaptic remodeling. However, not all combinations produce additive benefits; for instance, strength training combined with green tea supplementation yields only partial or context-dependent effects, highlighting that synergy is influenced by exercise type, compound specificity, and targeted cognitive domains (96). The absence of consistent additive effects across all intervention combinations highlights an important translational consideration. From the perspective of hormesis and the antioxidant paradox, excessive antioxidant exposure may attenuate exercise-induced adaptive signaling pathways that contribute to mitochondrial biogenesis, endogenous antioxidant defense, and neuronal plasticity. This mechanism may help explain why some studies report neutral, inconsistent, or only partially additive effects despite measurable improvements in oxidative stress markers. Low-to-moderate antioxidant supplementation may support redox homeostasis under conditions of excessive oxidative stress, whereas high-dose supplementation may excessively scavenge ROS required for exercise-induced mitohormesis and adaptive signaling. In addition, antioxidant administration immediately before or after exercise may interfere with ROS-dependent activation of pathways such as Nrf2 and mitochondrial biogenesis to a greater extent than appropriately timed or individualized supplementation strategies. Overall, current evidence indicates that exercise–antioxidant interactions are mechanistically pathway-specific rather than uniformly synergistic, involving selective convergence on redox regulation, neurotrophic signaling, autophagy, and neuroinflammatory pathways. Importantly, variability in outcomes across studies underscores the influence of exercise modality, exercise intensity and duration, antioxidant class, dosage, bioavailability, intervention timing, disease stage, and experimental model characteristics. These factors represent major sources of heterogeneity and highlight the need for precision-based intervention strategies that optimize antioxidant dose, timing, and exercise prescription according to disease stage and redox status.

**Table 3:** Overview of context-dependent effects of combined exercise and vitamin/antioxidant interventions on molecular pathways in experimental models of Alzheimer’s disease.

Population/model	Intervention	Dose	Duration	Mechanism	Findings	Exercise type	Evidence strength	Limitations/variability	Study
TMT-induced AD rats	Vitamin E	30 mg/kg	Not reported.	PI3K/NRF2 antioxidant pathway activation; reduced miR-125b, increased miR-132; decreased NF-κB; increased SOD, CAT; reduced MDA; increased angiogenesis	↓ Amyloid-β, ↓ NF-κB, ↓ MDA; ↑ SOD, ↑ CAT, ↑ PI3K, ↑ NRF2; ↓ miR-125b, ↑ miR-132; ↑ capillary density; improved hippocampal cell survival and memory	High-Intensity Interval Training (90–95% max speed)	Moderate (animal experimental study with mechanistic endpoints)	TMT model may not fully replicate AD; lack of precise reporting of intervention duration in abstract; translational gap to humans; intensity standardization issues. Potential synergy remains context-dependent and has not been compared with different antioxidant doses.	(83)
TMT-induced AD rats	Vitamin C	4 mg/kg	8 weeks	Reduced apoptosis signaling (↓ Caspase-3, FasL, Cyt-C, AP-1); shared anti-apoptotic pathway activation	↓ Caspase-3, ↓ FasL, ↓ Cyt-C, ↓ AP-1; strongest reduction in AT+VC group	Aerobic Training (8 weeks, 10–24 m/min)	Moderate	Limited to molecular apoptotic endpoints; no long-term behavioral follow-up; rodent-only model	(84)
TMT-induced AD rats	Vitamin C	4 mg/kg	8 weeks	Activation of PI3K/Nrf2 pathway; increased antioxidant enzymes (SOD, CAT); improved redox balance	↑ NRF2, ↑ SOD (all groups); ↑ Catalase (AT & AT+VC); ↑ PI3K and Catalase highest in AT+VC group	Aerobic Training (8 weeks, 10–24 m/min)	Moderate	ELISA-only protein quantification limits mechanistic depth; no behavioral correlation; variability in TMT severity	(85)
Aβ-induced AD rats	Coenzyme Q10	50 mg/kg	8 weeks	Reduction of oxidative stress (↓MDA, ↑TAC, ↑thiols, ↑GPx, ↑CAT); neuronal protection in hippocampus	↓ MDA; ↑ catalase, ↑ GPx, ↑ thiols; improved MWM & NORT performance; ↓ neuronal loss	HIIT (85–90% VO₂max intervals)	Moderate (controlled animal experiment, multi-arm design)	No dose–response assessment; whether the observed benefits exceed those of exercise alone remains uncertain.	(86)
Aβ-induced AD rats	Coenzyme Q10	50 mg/kg	8 weeks (pretreatment protocol)	Activation of FNDC5/irisin pathway → increased BDNF; reduced Aβ plaque formation	↑ FNDC5, ↑ irisin, ↑ BDNF; ↓ Aβ plaque formation	HIIT	Moderate	Biomarker-based (no electrophysiology); Aβ model limits disease complexity; no long-term cognitive follow-up beyond test window. Additive versus synergistic effects could not be clearly distinguished.	(87)
NSE/APPsw transgenic mice	Alpha-lipoic acid	100 mg/kg body weight/day	16 weeks	↑SOD-1, ↑CAT, ↑BDNF, ↑GLUT-1; oxidative stress reduction; partial neuroprotection	↑ BDNF, ↑ GLUT-1; ↑ SOD-1, ↑ CAT; improved memory; no significant change in Aβ-42 or apoptotic proteins	Treadmill exercise (16 weeks)	Moderate–high (long-duration transgenic model)	No synergistic effect on all markers (e.g., Bax, caspase-3 unchanged); no Aβ reduction despite functional improvement; incomplete dosing reporting	(88)
AlCl₃-induced AD rats	Zinc+ Melatonin	16 mg/kg zinc and 10 mg/kg melatonin	5 weeks	Modulation of GSK-3β/Wnt/β-catenin signaling; reduced oxidative stress & inflammation; reduced Aβ, APP, tau; improved neurotrophic support (BDNF)	↓ Aβ, ↓ Tau, ↓ APP; ↓ oxidative stress & inflammation; ↑ BDNF; improved cognition; improved liver & kidney function	Physical & Mental Activity (PMA)	Moderate	PMA not precisely quantified (intensity unclear); toxin-induced model differs from AD pathology; systemic (hepatorenal) effects complicate CNS interpretation. The relative contribution of exercise and supplementation to the observed benefits remains difficult to disentangle.	(89)

AD, Alzheimer’s disease; HIIT, high-intensity interval training; AT, aerobic training; VE, vitamin E; VC, vitamin C; CoQ10, coenzyme Q10; LA, alpha-lipoic acid; PMA, physical and mental activity; TMT, trimethyltin; Aβ, amyloid-beta; MWM, Morris water maze; NORT, novel object recognition test; NRF2, nuclear factor erythroid 2–related factor 2; PI3K, phosphoinositide 3-kinase; SOD, superoxide dismutase; CAT, catalase; GPx, glutathione peroxidase; MDA, malondialdehyde; NF-κB, nuclear factor kappa B; BDNF, brain-derived neurotrophic factor; FNDC5, fibronectin type III domain-containing protein 5; GLUT-1, glucose transporter 1; Cyt-C, cytochrome c; FasL, Fas ligand; AP-1, activator protein 1.

Note: The table is intended to present a neutral synthesis of the available evidence and does not assume a priori synergistic interactions between exercise and antioxidant supplementation. The terminology has been revised to avoid interpretative bias and to ensure descriptive consistency. While most studies report beneficial effects on cognitive and molecular outcomes in Alzheimer’s disease models, the evidence is not uniform, and the “antioxidant paradox” suggests that high-dose antioxidants may attenuate exercise-induced adaptive responses. In addition, a translational gap exists between robust effects observed in animal studies and more modest or inconsistent findings in human trials, potentially related to differences in bioavailability and blood–brain barrier constraints. Gut microbiota–related biomarkers were not consistently reported across the included studies and therefore were not incorporated into the table.

The magnitude and direction of the reported effects vary according to antioxidant type and dose, exercise modality and intensity, disease model, and treatment duration. Therefore, combined interventions should not be interpreted as uniformly synergistic across all experimental conditions.

**Table 4:** Overview of context-dependent effects of flavonoid and exercise interventions on cognitive function and molecular pathways in experimental models of Alzheimer’s disease.

Population/model	Intervention	Dose	Duration	Mechanism	Findings	Exercise type	Evidence strength	Limitations/variability	Study
STZ-induced AD rats	Quercetin	80 mg/kg	Quercetin: 21 days post-STZ; Exercise: 60 days	Reduction of oxidative stress in hippocampus; improved antioxidant defense system	↓ Oxidative stress; improved spatial memory; combination more effective than single treatments	Treadmill exercise (60 days, 20–22 m/min)	Moderate	STZ model does not fully replicate AD pathology; oxidative stress-focused endpoints; limited mechanistic depth (no pathway validation); additive versus synergistic effects were not clearly distinguished	(90)
TgCRND8 AD mice	EGCG	50 mg/kg/day	4 months	Reduction of soluble Aβ; modulation of amyloid burden; neurobehavioral improvement	↓ Aβ levels in cortex & hippocampus; improved learning, memory, and behavior (Barnes maze, nesting)	Voluntary wheel running (4 months)	Moderate–high (long-term intervention, behavioral + biochemical outcomes)	Voluntary exercise introduces variability in intensity; no precise workload quantification. The relative contribution of exercise and EGCG to the observed effects could not be clearly separated	(91)
Aβ1–42-induced AD mice	Luteolin	Not reported.	Not reported.	Modulation of autophagy pathways; correction of metabolic dysregulation (purine, retinol, thiamine, amino acid metabolism)	↑ Autophagy; improved energy metabolism; improved cognition (MWM); effects blocked by autophagy inhibitor (CQ)	Exercise (noted as combined intervention)	Moderate	Lack of dose and exercise intensity details; reliance on metabolomics (correlational); autophagy pathway inference indirect whether benefits reflect true synergy or pathway convergence remains uncertain	(92)
Aβ1–42-induced AD mice	Luteolin	100 mg/kg/day	Not reported.	Neuroinflammation reduction, autophagy enhancement	↓ Aβ; ↓ neuroinflammation; ↑ autophagy; improved cognition	Exercise	Moderate	Regional brain variability; incomplete exercise description; mechanistic confirmation indirect. Lack of dose–response assessment limits interpretation of interaction effects	(93)
Aluminium-induced neurotoxicity (zebrafish)	Apigenin	Not reported.	Chronic exposure	Antioxidant enzyme activation; anti-inflammatory effects	↑ antioxidant enzymes; ↑ AChE; ↓ neuroinflammation; improved behavior	Swimming-based physical activity	Low–moderate	Non-mammalian model; limited relevance to AD pathology; environmental toxin model rather than AD	(94)
APP/PS1 transgenic mice	Epicatechin	50 mg/kg/day	4 months	BDNF activation, Akt/GSK-3/CREB signaling, reduced Aβ	↓ Aβ; ↑ BDNF; ↓ oxidative stress; improved cognition	Treadmill (4 months)	High (preclinical consistency)	Strong animal evidence but limited human translation; genetic model constraints. Additive versus synergistic effects were not formally assessed.	(95)
Aβ-induced AD rats	Green tea (catechins)		8 weeks	Antioxidant activity, cholinergic modulation	Improved memory; ↑ AChE; mixed antioxidant effects; no strong synergy	Strength training (8 weeks)	Moderate	Some outcomes showed no synergism; variability in behavioral tests; peptide-based model limitations	(96)

AD, Alzheimer’s disease; STZ, streptozotocin; Aβ, amyloid-beta; EGCG, (−)-epigallocatechin-3-gallate; MWM, Morris water maze; NORT, novel object recognition test; AChE, acetylcholinesterase; BDNF, brain-derived neurotrophic factor; FNDC5, fibronectin type III domain-containing protein 5; CREB, cAMP response element-binding protein; Akt, protein kinase B; GSK-3, glycogen synthase kinase-3; CQ, chloroquine; APP/PS1, amyloid precursor protein/presenilin 1; Tg, transgenic; OR, object recognition; SR, social recognition.

Note: This table summarizes predominantly preclinical evidence and should therefore be interpreted cautiously because of important translational limitations. Although several studies report beneficial effects of combined flavonoid–exercise interventions, these findings should not be interpreted as evidence of uniform synergy across all experimental conditions. The magnitude and direction of responses appear to depend on flavonoid type and dose, exercise modality and intensity, disease model, intervention duration, and outcome measures. Consistent with the concept of the “antioxidant paradox,” excessive antioxidant exposure may attenuate exercise-induced adaptive signaling in certain contexts. Differences between robust preclinical findings and more modest clinical outcomes may also reflect limited bioavailability, blood–brain barrier permeability, and species-specific factors. In addition, gut microbiota-related biomarkers were rarely evaluated and represent an important gap in the current literature.

#### 6.1.4 Sources of heterogeneity and context-dependent neuroprotective effects

Overall, the evidence indicates that the neuroprotective effects of physical exercise alone or in combination with antioxidant or polyphenolic supplementation in AD models are highly heterogeneous and largely context-dependent. This variability arises from multiple interacting factors, including differences in exercise modality, intensity, and duration (aerobic exercise, HIIT, voluntary running, or multimodal training), as well as antioxidant characteristics such as type, dosage, route of administration, and bioavailability. In addition, species- and model-dependent differences (transgenic versus toxin-induced models), genetic background, disease stage, and methodological variables such as timing of intervention, disease stage, age, sex, and methodological variables such as timing of intervention, treatment duration, and metabolic state further contribute to divergent outcomes. Despite this heterogeneity, several convergent mechanistic pathways can be identified across studies, including activation of PI3K/NRF2-mediated antioxidant signaling, upregulation of BDNF/FNDC5-related neurotrophic pathways, modulation of NF-κB-dependent neuroinflammation, and regulation of apoptotic, autophagic, and amyloid-related processes. These shared pathways suggest partial mechanistic convergence underlying neuroprotection, although the extent of engagement varies substantially across interventions. An additional source of variability may relate to differences in adaptive redox signaling across experimental conditions. Exercise-induced increases in ROS are increasingly recognized as important signaling events that activate endogenous antioxidant defenses, mitochondrial biogenesis, autophagy, and neurotrophic pathways. The extent of this effect is likely influenced by antioxidant dosage and timing of administration. Low-to-moderate antioxidant supplementation may support redox homeostasis under conditions of excessive oxidative stress, whereas high-dose or inappropriately timed supplementation may excessively scavenge ROS required for exercise-induced mitohormesis, mitochondrial biogenesis, and endogenous antioxidant responses. This hormetic framework provides a plausible explanation for some of the neutral, inconsistent, or attenuated effects reported across studies. Importantly, these observations suggest that successful neuroprotection depends not only on reducing oxidative damage but also on preserving the adaptive redox signaling required for endogenous cellular defense and neuronal resilience. Collectively, these findings support a framework in which exercise–antioxidant interactions confer neuroprotection through selective and context-dependent convergence of redox, neurotrophic, mitochondrial, autophagic, and inflammatory pathways rather than through a uniform biological response. This perspective has important translational implications, emphasizing the need to optimize intervention design, antioxidant dose and timing, and exercise prescriptions according to disease stage, redox status, and molecular context to maximize therapeutic efficacy in AD.

### 6.2 PD models

#### 6.2.1 Effect of exercise alone

Exercise interventions in PD produce heterogeneous outcomes; nevertheless, many studies suggest convergence on several common neuroplastic and neurotrophic mechanisms. Across studies, three major adaptive domains can be identified: (i) neurotrophic upregulation and synaptic plasticity, (ii) large-scale motor network reorganization, and (iii) intensity- and protocol-dependent variability in systemic biomarkers. A central and highly consistent mechanism involves upregulation of neurotrophic signaling, particularly BDNF. Moderate-intensity aerobic interval training, resistance training, Nordic walking, and high-intensity cycling all demonstrate increases in circulating BDNF, often accompanied by improved motor performance and functional outcomes (97–100) (Table 5). Collectively, these findings suggest that exercise-induced neuroprotection in PD is mediated by coordinated activation of neurotrophic, metabolic, and redox-sensitive pathways rather than by isolated changes in individual biomarkers. The convergence of BDNF, FNDC5/irisin, and AMPK-related signaling pathways highlights a broader adaptive network linking peripheral physiological responses to central nervous system plasticity.

**Table 5:** Overview of exercise interventions and context-dependent neuroplasticity-related outcomes in Parkinson’s disease.

Population/model	Intervention/exercise type	Duration	Mechanism	Findings	Evidence strength	Limitations/variability	Study
Human PD	Aerobic Interval Training (AIT)	12 weeks	Enhances neuroplasticity via BDNF modulation in M1 and SMA	↑ BDNF, ↑ motor function; EEG changes; ↔ [18F]DOPA PET	Moderate–Strong (Human RCT; consistent motor/BDNF improvements but no dopaminergic PET change)	No change in dopaminergic PET imaging; small sample size; peripheral BDNF may not reflect CNS levels; short duration (12 weeks). Inter-individual variability in neuroplastic responses was not systematically explored	(97)
Human PD	Home-Based High-Intensity Interval Training (HIIT)	12 weeks	Insufficient intensity to induce neuroplastic changes	↑ Feasibility, adherence 78.4%, safe; ↔ BDNF, VO₂max, UPDRS III	Weak–Moderate (Feasibility RCT; no significant neurotrophic or clinical improvement)	Did not achieve target intensity in all participants; small sample (*n* = 13); adherence variability; feasibility-focused design not efficacy-powered	(104)
Human PD	Multimodal Exercise components were combined, making the relative contribution of each modality difficult to determine	12 weeks	Promotes neurotrophic support and neuroplasticity	↑ BDNF, ↑ GDNF	Moderate (Quasi-experimental human study with positive neurotrophic changes)	Non-RCT design; moderate sample size; peripheral biomarkers only; limited CNS mechanistic confirmation	(98)
Human PD	HIIT and Continuous Aerobic Exercise (CAE)	HIIT duration: 4 weeks CAE duration: 4 weeks	Exercise modulates motor function and neuroplasticity markers, responses are individualized	Improved motor symptoms; heterogeneous individual responses; non-motor symptoms variable	Weak–Moderate (Single-case experimental design with high variability and limited generalizability)	Very small sample size (*n* = 3); marked inter-individual variability; no consistent superiority of HIIT or CAE; drop-out limited within-subject comparisons; limited external validity	(106)
MPTP-induced PD mice	Treadmill Exercise	8 weeks	Activates FNDC5-BDNF pathway; strengthens hippocampal synaptic plasticity and nigro-hippocampal dopaminergic projections	↑ FNDC5, ↑ BDNF, ↑ dopamine; improved cognition and motor function	Strong (Preclinical mechanistic evidence with robust molecular effects)	Limited translational applicability to humans; MPTP model does not fully replicate human PD pathology; controlled experimental conditions reduce ecological validity	(101)
Human PD	Nordic Walking (NW)	5 months	Aerobic exercise elevates BDNF, enhancing motor function and neuroplasticity	↑ gait, ↑ motor function, ↑ daily steps; ↑ serum BDNF; sustained improvements	Moderate (Prospective human study with functional and biomarker improvements)	Small sample size; lack of randomized control group; heterogeneity in disease severity; serum BDNF as a peripheral surrogate marker; non-motor outcomes not statistically significant	(99)
PD mice	Resistance Exercise	5 weeks	AMPK phosphorylation upregulates BDNF and neurogenesis, enhancing spatial learning	↑ coordination, ↑ spatial learning, ↑ BDNF, ↑ TrkB, ↑ AMPK phosphorylation	Strong (Preclinical mechanistic evidence with consistent molecular and behavioral improvements in animal model)	Animal model findings may not fully translate to human PD; controlled experimental conditions limit clinical applicability; no long-term translational validation	(102)
Human PD	Task-Oriented + Aerobic Training (TOT-AT)	8 weeks	TOT-AT insufficient to induce measurable neurotrophic or anti-inflammatory responses	No significant change in serum BDNF, GDNF, IGF-1, VEGF, TNF-α, IL-1β	Weak–Moderate (Randomized controlled study with negative biomarker findings)	Lack of biomarker response despite intervention; relatively short intervention period; serum biomarkers may not accurately reflect central neuroplasticity; modest sample size after study completion	(105)
Human PD	High-Intensity Tandem Bicycle whether similar responses occur in advanced PD stages remains uncertain	16 weeks	High-intensity aerobic exercise elevates BDNF, improving motor function and neural network activation	↑ BDNF (>10-fold), ↑ VO₂max, ↓ UPDRS, improved fMRI connectivity	Moderate–Strong (Human clinical intervention with biomarker and neuroimaging support)	Small sample size; single-center study; short-term follow-up; plasma BDNF as indirect CNS marker; preliminary neuroimaging findings require further validation	(100)
Human PD	HIIT vs. Moderate-Intensity Continuous Training (MICT)	12 weeks	High-intensity training preferentially induces BDNF, promoting neuroplasticity	↑ BDNF in HIIT, not MICT	Moderate (Pilot human study demonstrating intensity-dependent neurotrophic response)	Pilot study with limited sample size; lack of direct neuroplasticity imaging measures; unclear long-term clinical relevance; variability in individual exercise responses	(103)

BDNF, brain-derived neurotrophic factor, GDNF, glial cell line-derived neurotrophic factor, FNDC5, fibronectin type III domain-containing protein 5, M1, primary motor cortex, SMA, supplementary motor area, UPDRS, Unified Parkinson’s Disease Rating Scale, EEG, electroencephalography, fMRI, functional magnetic resonance imaging, VO₂max, maximal oxygen uptake, HIIT, high-intensity interval training, MICT, moderate-intensity continuous training, TOT-AT, task-oriented plus aerobic training.

The magnitude of exercise-induced neuroplastic adaptations varies substantially according to exercise modality, intensity, duration, disease severity, baseline functional status, and biomarker assessment methods. Therefore, the reported findings should not be interpreted as uniform responses across all Parkinson’s disease populations or exercise protocols.

In experimental models, exercise further activates FNDC5/irisin signaling, which enhances hippocampal dopamine availability and promotes BDNF expression through integrin-mediated pathways, indicating a muscle–brain endocrine axis that links peripheral exercise responses to central neuroplasticity (101). Resistance training additionally activates AMPK phosphorylation, which further enhances BDNF/TrkB signaling and promotes neurogenesis, suggesting that energy-sensing pathways play a critical role in mediating exercise-induced neuroplasticity (102). A second mechanistic theme involves functional and network-level reorganization of motor circuits. EEG and neuroimaging studies indicate that exercise enhances resting-state synchronization in the primary motor cortex and promotes large-scale motor network reorganization, which correlates with improvements in motor performance (97, 100). Importantly, these functional changes may occur independently of dopaminergic terminal integrity, as no significant alterations in PET/CT dopaminergic uptake were observed following aerobic interval training, suggesting that exercise primarily exerts compensatory neuroplastic rather than structural dopaminergic effects (97). The association between increased BDNF levels, cortical synchronization, and improved Unified Parkinson’s Disease Rating Scale scores further supports a neurotrophic–network coupling mechanism underlying motor recovery (100). A third important domain is intervention heterogeneity and intensity-dependent responsiveness. Not all exercise protocols produce uniform neurobiological effects. HIIT and multimodal interventions combining resistance, aerobic, and balance training generally produce stronger neurotrophic responses compared with single-modality or moderate interventions (98, 103). However, feasibility studies show that HIIT may not consistently increase BDNF or improve clinical scores in all cohorts, likely due to sample size limitations, disease stage, or insufficient adaptation time (104). Similarly, short-duration multimodal rehabilitation may fail to induce measurable changes in systemic neurotrophic or inflammatory biomarkers despite structured training (105). These findings highlight that exercise-induced adaptations are highly dependent on exercise intensity, modality, intervention duration, baseline disease severity, medication status, and individual physiological responsiveness. Importantly, the observed heterogeneity across exercise studies may also reflect differences in the magnitude of adaptive stress responses induced by specific training protocols. Exercise-mediated increases in ROS are increasingly recognized as physiological signals that promote mitochondrial adaptation, endogenous antioxidant defenses, and neuroplasticity. Consequently, variations in exercise intensity and training load may influence the degree of activation of these protective pathways.

**Figure 3:**
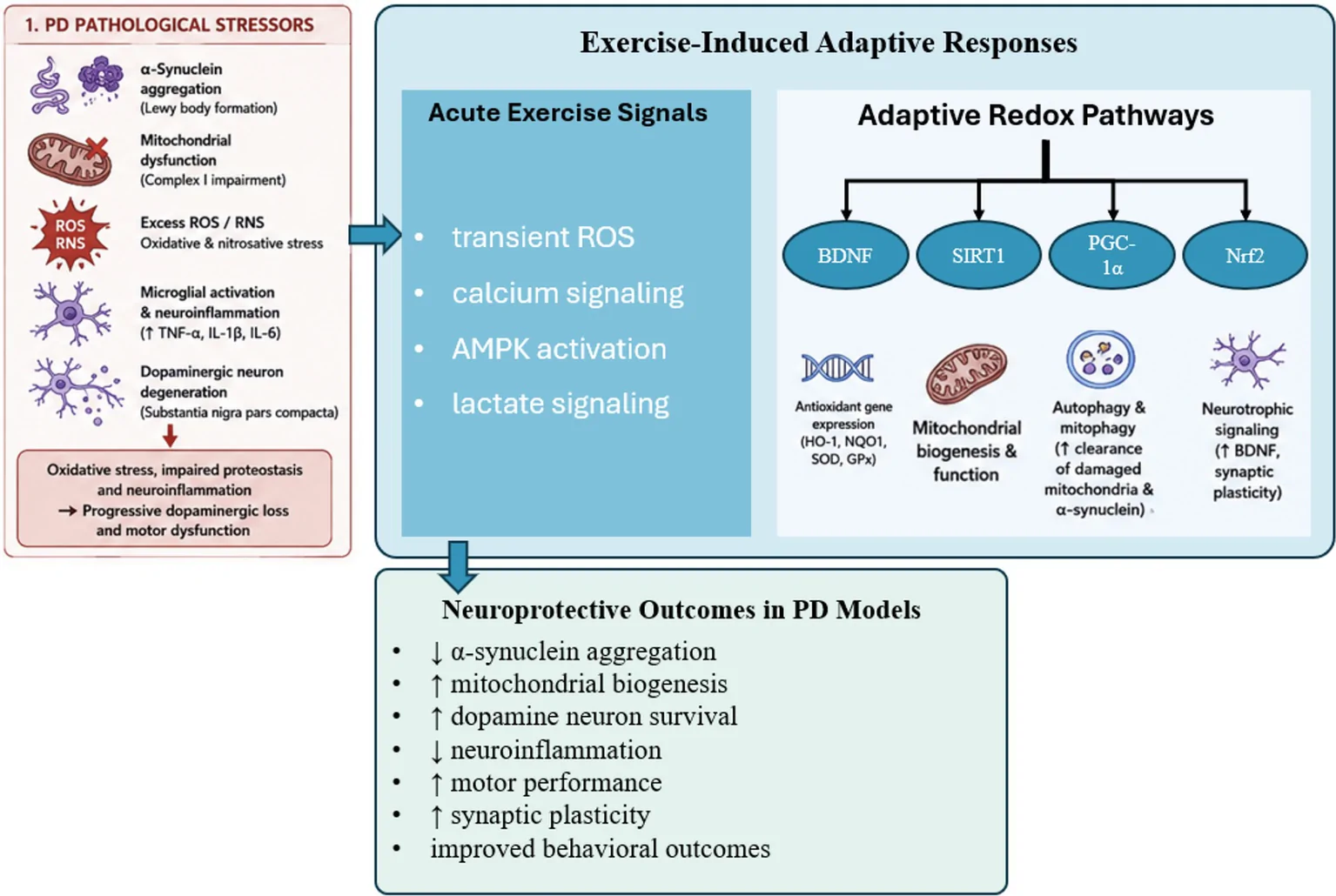
Exercise–redox–antioxidant interactions in experimental Parkinson’s disease (PD) models.

In addition, significant inter-individual variability is observed in response to exercise interventions. Crossover and pilot studies demonstrate that motor and biomarker responses vary substantially between individuals, with no consistent superiority of a single exercise modality such as HIIT versus continuous aerobic training (106). These observations are also relevant to the concept of hormesis, whereby moderate physiological stress elicits adaptive biological responses. Accordingly, excessive or inappropriately timed antioxidant supplementation could theoretically attenuate exercise-induced adaptive signaling by reducing ROS-dependent activation of neuroprotective pathways. The extent of this interaction is likely influenced by antioxidant dosage, timing of administration, and the underlying redox status of the individual. This concept provides a mechanistic framework for interpreting studies in which combined exercise–antioxidant interventions fail to produce additive benefits. Collectively, current evidence indicates that exercise primarily improves motor and functional outcomes in PD through coordinated activation of BDNF-mediated neuroplasticity, FNDC5–BDNF signaling, metabolic adaptation, and large-scale motor network reorganization rather than through direct restoration of dopaminergic degeneration. However, the magnitude of these adaptations is strongly influenced by exercise intensity, modality, intervention duration, disease stage, medication status, and inter-individual variability. Together, these factors represent major sources of heterogeneity across studies and should be considered when designing personalized exercise interventions for PD. These factors represent important sources of heterogeneity and have direct translational implications, supporting the development of personalized exercise prescriptions to maximize therapeutic efficacy in PD rehabilitation (Figure 3).

The figure illustrates the mechanistic interplay among Parkinson’s disease pathology, exercise-induced adaptive signaling, and antioxidant modulation in experimental PD models. PD-related stressors, including *α*-synuclein aggregation, mitochondrial dysfunction, excessive ROS/RNS production, neuroinflammation, and dopaminergic neuronal degeneration, contribute to oxidative stress, impaired proteostasis, and progressive motor dysfunction. Exercise induces transient physiological ROS generation and metabolic stress, which activate adaptive redox-sensitive pathways including Nrf2, AMPK, SIRT1, PGC-1*α*, and BDNF signaling. These hormetic responses may enhance antioxidant defense, mitochondrial biogenesis, autophagy/mitophagy, neuroplasticity, and cellular resilience, thereby potentially reducing α-synuclein pathology, neuroinflammation, and dopaminergic neuronal loss while improving motor and cognitive outcomes. The figure also highlights the “antioxidant paradox,” whereby moderate ROS signaling promotes beneficial mitohormetic adaptations, whereas excessive antioxidant supplementation may suppress adaptive redox signaling and attenuate exercise-induced neuroprotective responses. Importantly, interactions between exercise and antioxidant supplementation are context-dependent rather than uniformly synergistic, with outcomes varying according to antioxidant type and dose, timing of supplementation, exercise modality and intensity, disease stage, experimental model, and baseline redox status. Accordingly, combined interventions may produce additive, synergistic, neutral, or even attenuated effects under different conditions. Lower panels summarize the major pathological features and exercise-related findings across common PD experimental models, including MPTP/MPP+, 6-OHDA, rotenone, and *α*-synuclein transgenic models. The figure represents a conceptual framework and does not imply universally consistent biological responses across all PD models or clinical settings.

#### 6.2.2 Effect of antioxidant supplementation alone

Antioxidant supplementation in PD models and clinical settings produces modest and highly heterogeneous effects, with outcomes largely reflecting indirect modulation of cellular resilience, mitochondrial function, synaptic signaling, and gut–brain axis interactions rather than direct dopaminergic restoration. Across studies, four main mechanistic patterns emerge: (i) partial compensatory postsynaptic signaling modulation, (ii) mitochondrial and oxidative stress buffering, (iii) prophylactic versus therapeutic stage-dependent efficacy, and (iv) microbiota-mediated neuroprotection. A first mechanistic theme involves limited postsynaptic and compensatory signaling effects without true neuroprotection. Vitamin A supplementation in 6-OHDA models improves voluntary motor activity but does not prevent dopaminergic neuron loss or dopamine depletion. The observed upregulation of D2 and RXR receptors suggests that vitamin A primarily modulates postsynaptic signaling pathways, reflecting compensatory adaptations rather than structural neuroprotection (107). This indicates that functional improvements may occur independently of disease modification at the nigrostriatal level. A second consistent mechanism is mitochondrial stabilization and oxidative stress attenuation, particularly with CoQ10 supplementation. In both clinical and experimental PD models, CoQ10 provides modest improvements in motor and visual function and reduces oxidative damage markers such as protein carbonyls, suggesting enhancement of mitochondrial resilience and antioxidant buffering capacity (108, 109). Importantly, its effects are more pronounced when administered preventively rather than therapeutically, indicating that CoQ10 primarily enhances neuronal resistance to oxidative insults rather than reversing established neurodegeneration (109). Importantly, the stage-dependent effects observed with antioxidant interventions suggest that oxidative stress is not merely a pathological process but may also participate in adaptive cellular signaling. The magnitude of this effect is likely further influenced by antioxidant dosage, timing of administration, and disease stage. Low-to-moderate antioxidant supplementation may support mitochondrial function and redox homeostasis under conditions of excessive oxidative stress, whereas high-dose or prolonged supplementation could potentially attenuate adaptive redox signaling pathways required for endogenous cellular defense and stress adaptation.

A third mechanistic domain involves non-cell-autonomous and microbiota-dependent neuroprotection. EGCG in a PINK1-mutant Drosophila PD model improves motor performance, survival, and mitochondrial integrity through modulation of gut microbiota composition. The attenuation of neuroprotective effects following microbiota disruption, along with the identification of strain-specific mediators such as TotM, indicates that EGCG acts primarily through a gut–brain axis mechanism rather than direct neuronal antioxidant activity (110). The mechanistic diversity observed across antioxidant interventions further highlights that neuroprotection in PD is unlikely to be achieved through a single antioxidant pathway. Instead, effective responses appear to depend on the coordinated modulation of mitochondrial function, cellular stress responses, neuroinflammation, and gut–brain communication, which may explain the variability reported across experimental models and clinical studies. Additional sources of heterogeneity include differences in antioxidant class, dosage, bioavailability, treatment duration, disease stage, genetic background, and experimental model characteristics. From a translational perspective, these findings suggest that antioxidant monotherapy is unlikely to provide consistent disease-modifying effects across all stages of PD. Future strategies may benefit from combining antioxidant interventions with exercise and other lifestyle approaches that engage complementary neuroprotective pathways while preserving adaptive redox signaling; however, the efficacy of such combined interventions is likely to remain highly context-dependent.

#### 6.2.3 Effect of combined exercise and antioxidants

In a reserpine-induced PD model, combined vitamin E supplementation and aerobic exercise improved mitochondrial homeostasis by modulating key regulators of mitochondrial dynamics and quality control. Specifically, the intervention reduced cytochrome c expression while increasing Pink1, ATP synthase, and Opa1 levels, suggesting enhanced mitophagy, energy production, and mitochondrial fusion processes. However, the absence of change in 8-oxodeoxyguanosine indicates that systemic DNA oxidative damage may not be substantially affected by this combined intervention, implying a selective rather than global antioxidant effect (111). Although evidence regarding combined exercise–antioxidant interventions in PD remains limited, available findings suggest that improvements in mitochondrial quality control may represent one important mechanism underlying neuroprotection. The selective modulation of mitophagy, mitochondrial fusion, and energy metabolism observed in this model supports the broader concept that neuroprotection is mediated through pathway-specific adaptations rather than generalized antioxidant effects. These observations are also consistent with the concept of hormesis, whereby preservation of adaptive redox signaling may be necessary to trigger mitochondrial remodeling and cellular stress-response pathways. Additional studies are required to determine whether antioxidant supplementation enhances or attenuates exercise-induced neuroprotective adaptations under different experimental conditions. In particular, the effects of antioxidant dosage, timing of administration, treatment duration, and disease stage require further investigation, as excessive or inappropriately timed antioxidant supplementation may interfere with ROS-dependent adaptive signaling involved in mitochondrial remodeling and neuroplasticity.

#### 6.2.4 Sources of heterogeneity and context-dependent neuroprotective responses in PD models

Evidence from PD studies demonstrates substantial heterogeneity in the neuroprotective effects of exercise performed alone or in combination with antioxidant supplementation. This variability is largely attributable to differences in exercise modality, intensity, and duration, together with inconsistencies in antioxidant type, dosage, timing, route of administration, and bioavailability. Additional sources of heterogeneity include disease stage, medication status, age, sex, genetic background, and differences between toxin-induced and genetic PD models. Species-specific and model-dependent factors, such as those associated with toxin-induced versus genetic PD models, are likely to contribute to the observed discrepancies in pathological and behavioral outcomes. It is noteworthy that outcome measures themselves differ in sensitivity and specificity, with behavioral enhancements frequently occurring in the absence of consistent biomarker or neurochemical alterations. An additional source of heterogeneity may relate to differences in adaptive redox responses across interventions. Exercise-induced increases in ROS are increasingly recognized as physiological signals that activate endogenous antioxidant defenses, mitochondrial biogenesis, and neuroplasticity-related pathways. The magnitude of this interaction is likely influenced by antioxidant dosage and timing of administration. Low-to-moderate antioxidant supplementation may help restore redox homeostasis under conditions of excessive oxidative stress, whereas high-dose or inappropriately timed supplementation may excessively suppress ROS-dependent signaling pathways required for mitochondrial biogenesis, endogenous antioxidant defense, and neuroplastic adaptation. Collectively, these findings indicate that exercise–antioxidant interventions do not produce uniform biological responses in PD. Instead, neuroprotective effects appear to emerge through context-dependent interactions among neurotrophic, mitochondrial, inflammatory, and redox-sensitive pathways. This framework helps explain the heterogeneous outcomes reported across studies and highlights the importance of optimizing intervention design, antioxidant dosing, exercise prescription, and disease-stage selection to improve translational relevance.

## 7 Comparative effects: combination vs. monotherapy

A growing body of evidence suggests that both physical exercise and antioxidant supplementation independently confer neuroprotective effects; however, their combined application yields heterogeneous outcomes depending on intervention timing, antioxidant dosage, disease stage, and redox context. Exercise enhances neuroplasticity, mitochondrial biogenesis, and endogenous antioxidant defenses through controlled ROS-mediated hormetic signaling, whereas antioxidant supplementation reduces oxidative stress and inflammatory signaling but may also interfere with adaptive redox responses. In this context, several inconsistent findings across studies may be better understood through the framework of the antioxidant paradox and hormesis theory. For instance, although combined interventions frequently improve cognitive performance, Aβ burden, and antioxidant enzyme activity, some studies report no additional benefit over exercise alone. Such observations may reflect attenuation of ROS-dependent signaling required for full activation of adaptive pathways such as Nrf2 and BDNF, rather than a lack of biological efficacy per se (83–85). Mechanistically, synergistic effects reported in studies involving PI3K/NRF2 activation, BDNF upregulation, and autophagy modulation suggest convergence on redox-sensitive signaling networks. However, the magnitude of these effects appears highly dependent on antioxidant dose and timing. Low-to-moderate antioxidant supplementation may support redox homeostasis under conditions of excessive oxidative stress, whereas high-dose or inappropriately timed supplementation may excessively scavenge ROS and thereby blunt exercise-induced mitochondrial, neurotrophic, and synaptic adaptations (86–88). Importantly, a consistent pattern emerging across both AD and PD models is that interventions targeting multiple interconnected pathways—including redox regulation, mitochondrial function, neurotrophic signaling, autophagy, and neuroinflammation—tend to produce more robust neuroprotective effects than strategies focused solely on oxidative stress reduction. This observation supports the concept that successful neuroprotection requires preservation of adaptive cellular signaling in addition to limiting oxidative damage. Conversely, in conditions where antioxidant supplementation remains within physiological thresholds, additive or synergistic improvements in oxidative stress markers and cognitive outcomes are observed. The substantial variability observed across studies likely reflects differences in antioxidant class, dosage, timing of administration, exercise modality, intervention duration, baseline oxidative status, disease stage, genetic background, and methodological characteristics of experimental models and clinical populations. These factors represent major sources of heterogeneity and may explain why apparently similar interventions produce divergent outcomes across experimental models and clinical populations. Collectively, these findings highlight that the interaction between exercise and antioxidant supplementation is not strictly additive but rather governed by redox balance thresholds, supporting a context-dependent and dose-sensitive model of neuroprotection. From a translational perspective, these observations suggest that the goal of combined interventions should not be the maximal suppression of ROS but the optimization of redox homeostasis while preserving exercise-induced adaptive signaling. Future clinical studies should therefore focus on identifying antioxidant dosing strategies, timing schedules, and exercise prescriptions that maximize complementary neuroprotective mechanisms without impairing hormetic adaptations.

## 8 Translational and clinical perspectives

Translational application of combined exercise and antioxidant strategies is limited by pharmacokinetic constraints, variability in exercise protocols, and inter-individual differences in redox status. While clinical and preclinical studies consistently demonstrate benefits of exercise on motor function and cognition, antioxidant supplementation yields more variable outcomes. Importantly, several negative or null findings including lack of significant improvement in neurotrophin levels or absence of additional benefit from combined interventions, may be interpreted through the lens of the antioxidant paradox. In such cases, excessive antioxidant intake may suppress ROS-dependent adaptive signaling pathways required for exercise-induced neuroplasticity, thereby blunting expected increases in neurotrophic factors rather than indicating true therapeutic inefficacy (97–106). This interpretation is consistent with the concept of hormesis, whereby moderate increases in ROS act as physiological signals that trigger endogenous antioxidant defenses, mitochondrial biogenesis, autophagy, and neurotrophic adaptations. The magnitude of this effect is likely influenced by antioxidant dosage and timing of administration. Low-to-moderate antioxidant supplementation may support redox homeostasis under conditions of excessive oxidative stress, whereas high-dose or inappropriately timed supplementation may excessively scavenge ROS required for exercise-induced mitohormesis, mitochondrial biogenesis, and neuroplastic adaptation. Similarly, variability in responses across intervention studies, including pharmacological–exercise interaction paradigms, may reflect interference with redox-sensitive signaling cascades essential for mitochondrial biogenesis and synaptic remodeling. More broadly, current evidence suggests that successful neuroprotection depends not only on reducing oxidative damage but also on preserving adaptive redox signaling. This concept may explain why interventions producing measurable improvements in oxidative biomarkers do not always translate into proportional functional or cognitive benefits. These findings emphasize that optimal therapeutic outcomes depend not only on combining interventions but on preserving physiological hormetic signaling. From a translational standpoint, additional limitations include poor blood–brain barrier (BBB) permeability, efflux transporter activity (e.g., P-glycoprotein), and rapid metabolic degradation of many antioxidant compounds (112–118). These factors further complicate achieving effective central nervous system concentrations. Furthermore, substantial heterogeneity across studies arises from differences in antioxidant class, dosage, timing of administration, bioavailability, exercise modality, intervention duration, disease stage, baseline redox status, genetic background, medication use, and methodological characteristics. These factors should be considered when interpreting conflicting findings and designing future clinical trials. Overall, successful clinical translation requires a precision-based approach in which antioxidant dosing, timing relative to exercise, and individual redox status are carefully optimized. Excessive antioxidant supplementation may, under certain circumstances, be counterproductive, whereas appropriately calibrated interventions may preserve hormetic signaling while reducing pathological oxidative stress. Future clinical investigations should prioritize biomarker-guided intervention strategies, standardized exercise protocols, and dose- and timing-optimization studies to identify therapeutic windows that maximize complementary neuroprotective effects while minimizing interference with adaptive exercise-induced signaling pathways.

## 9 Emerging concepts and future directions

Recent advances have identified several emerging concepts that may improve our understanding of how exercise and antioxidant interventions interact in NDDs. The epigenetic regulation, encompassing DNA methylation, histone modifications, and microRNAs (miRNAs), assumes a pivotal role in the modulation of neuronal gene expression and redox-sensitive pathways. Empirical evidence indicates that exercise and antioxidant supplementation can affect these epigenetic mechanisms, thereby potentially reinstating adaptive transcriptional programs that mitigate the effects of neurodegeneration. For example, particular miRNAs associated with oxidative stress, inflammation, and neuroplasticity may be modulated through combined interventions, thereby presenting novel targets for precision therapy. The gut–brain axis represents another promising domain of inquiry. The composition of gut microbiota and their metabolite profiles exert influence over neuroinflammation, oxidative stress, and neurotransmitter signaling pathways. Emerging data suggest that dietary antioxidants and physical activity may act synergistically to modulate the gut microbiome, thereby enhancing neuroprotective metabolites such as SCFAs while concurrently diminishing pro-inflammatory signals. This bidirectional communication facilitates the exploration of microbiota-targeted interventions as integral components of multimodal strategies aimed at promoting brain health. Personalized medicine methodologies are anticipated to optimize the efficacy of combination therapies. Individual variability in genetic, epigenetic, lifestyle factors, and disease progression can significantly influence responses to exercise and antioxidant interventions. Additional sources of variability, including differences in baseline redox status, medication use, sex, age, and intervention characteristics, may further contribute to heterogeneous treatment responses and should be considered when developing personalized therapeutic strategies. Customizing interventions based on biomarkers indicative of oxidative stress, neuroinflammation, and mitochondrial functionality may enhance therapeutic outcomes while concurrently mitigating potential adverse effects. An important future challenge will be defining the optimal balance between reducing pathological oxidative stress and preserving adaptive redox signaling. Increasing evidence from exercise biology suggests that ROS function not only as mediators of cellular damage but also as critical signaling molecules that regulate mitochondrial biogenesis, endogenous antioxidant defenses, autophagy, and neuroplasticity. Future studies should therefore investigate how antioxidant type, dosage, timing, and bioavailability influence hormetic adaptations and whether individualized antioxidant strategies can maximize neuroprotection without attenuating exercise-induced benefits. Particular attention should be given to determining whether low-to-moderate antioxidant supplementation supports adaptive responses, whereas high-dose or inappropriately timed supplementation may suppress ROS-dependent signaling required for exercise-induced mitochondrial remodeling and neuroplasticity. Defining these therapeutic windows will be essential for optimizing combined intervention strategies.

The integration of advanced computational and technological approaches, including artificial intelligence and machine learning algorithms, may facilitate individualized optimization of exercise prescriptions by analyzing multidimensional patient data such as physiological responses, activity patterns, and disease progression trajectories. In parallel, multi-omics approaches (genomics, transcriptomics, proteomics, and metabolomics) may provide a more comprehensive understanding of disease heterogeneity and treatment response variability. Such approaches may also help identify patient subgroups that are most likely to benefit from specific exercise–antioxidant combinations, thereby improving translational success and reducing inconsistencies observed across clinical studies. Ultimately, multi-target therapeutic frameworks that amalgamate exercise, dietary antioxidants, cognitive training, and pharmacological agents signify a progressive paradigm. By concurrently addressing oxidative stress, mitochondrial dysfunction, neuroinflammation, and proteostasis, such strategies may provide broader neuroprotective benefits by simultaneously targeting oxidative stress, mitochondrial dysfunction, neuroinflammation, and proteostasis. Translational advancements in nanocarrier-based drug delivery systems and BBB-targeted strategies may significantly enhance the bioavailability and central nervous system penetration of antioxidant compounds, thereby overcoming one of the major limitations in clinical translation. Ongoing investigation into these interrelated pathways will be imperative for the translation of preclinical insights into precision-driven, clinically relevant interventions. Particular attention should be given to determining whether low-to-moderate antioxidant supplementation supports adaptive responses, whereas high-dose or inappropriately timed supplementation may suppress ROS-dependent signaling required for exercise-induced mitochondrial remodeling and neuroplasticity.

## 10 Limitations of current evidence

Despite encouraging findings, several limitations constrain the interpretation and translational applicability of current evidence regarding exercise and antioxidant interventions in NDDs. One of the most important challenges is the gap between experimental models and human disease. Although animal models provide valuable mechanistic insights into redox signaling, neuroplasticity, autophagy, and mitochondrial dynamics, they do not fully capture the complexity of human NDDs, including variability in disease onset, progression, genetic background, lifestyle factors, and comorbidities. Consequently, promising preclinical findings do not always translate into consistent clinical benefits. Another major limitation is the substantial heterogeneity of intervention protocols. Differences in exercise modality, intensity, duration, and frequency, together with variability in antioxidant type, dosage, bioavailability, and timing of administration, complicate comparisons across studies and limit the establishment of clear dose–response relationships. As a result, the identification of optimal intervention parameters and the development of standardized clinical recommendations remain challenging. Additional sources of heterogeneity include differences in animal strains, genetic background, sex, age, disease models, and outcome measures in preclinical studies, as well as variability in disease stage, medication use, lifestyle characteristics, baseline physiological status, and baseline redox status in clinical populations. These factors contribute substantially to inconsistencies across studies and may partly explain the divergent findings reported for both exercise and antioxidant interventions. Furthermore, relatively few studies have systematically evaluated combined exercise–antioxidant interventions while adequately controlling for potential confounding factors. This limitation makes it difficult to distinguish true synergistic effects from additive, neutral, or potentially antagonistic interactions. Another important consideration is the concept of hormesis and the so-called “antioxidant paradox,” which may provide a mechanistic explanation for some of the inconsistent findings observed across experimental and clinical investigations. Moderate increases in ROS generated during exercise can activate adaptive signaling pathways involved in mitochondrial biogenesis, Nrf2-mediated antioxidant responses, autophagy, and neuroplasticity. In contrast, excessive antioxidant supplementation may suppress these beneficial redox-sensitive signaling processes. The magnitude of this effect is likely influenced by antioxidant dose, timing of administration, and disease context. Low-to-moderate antioxidant supplementation may support redox homeostasis under conditions of excessive oxidative stress, whereas high-dose or inappropriately timed supplementation may excessively scavenge ROS required for exercise-induced mitohormesis and neuroplastic adaptation. Accordingly, the absence of expected benefits in some intervention studies should not necessarily be interpreted solely as evidence of therapeutic failure. Rather, such findings may reflect disruption of exercise-induced adaptive signaling due to inappropriate antioxidant dosing, timing, or intervention design. This perspective may help explain why improvements in biochemical markers of oxidative stress do not always translate into measurable functional or cognitive outcomes. Taken together, these limitations underscore the need for more standardized study designs, adequately powered clinical trials, biomarker-guided intervention strategies, and careful consideration of redox biology when evaluating combined exercise–antioxidant approaches. Addressing these challenges will be essential for improving reproducibility, reducing heterogeneity, and facilitating successful clinical translation.

## 11 Conclusion

In conclusion, accumulating evidence suggests that physical exercise and antioxidant systems interact through multiple redox-sensitive mechanisms that may influence key pathological processes underlying NDDs, particularly AD and PD. Collectively, current findings suggest that exercise and antioxidant interventions converge on common molecular pathways, including Nrf2-mediated antioxidant defense, BDNF-dependent neuroplasticity, autophagy regulation, and mitochondrial biogenesis and dynamics. These shared mechanisms provide a plausible framework through which combined interventions may promote neuronal resilience and cognitive health. Although many studies report greater benefits from combined exercise-antioxidant approaches than from either intervention alone, findings remain heterogeneous across experimental models and clinical settings. Variability in antioxidant type, dosage, timing of administration, exercise modality, intensity, and disease stage may contribute to these differences. Importantly, emerging evidence suggests that excessive, high-dose, or inappropriately timed antioxidant supplementation may attenuate exercise-induced adaptive redox signaling, potentially limiting neuroprotective responses and contributing to the neutral or inconsistent outcomes reported in certain studies. Despite encouraging progress, much of the available evidence remains preclinical, underscoring the need for well-designed clinical trials to establish efficacy, optimize intervention protocols, and identify patient-specific strategies. Future research should also clarify the roles of epigenetic regulation, gut–brain axis signaling, and redox-sensitive molecular networks in mediating exercise-antioxidant interactions. However, the efficacy of combined exercise–antioxidant interventions is likely to remain highly context-dependent and influenced by factors such as antioxidant dose, timing of administration, disease stage, and individual redox status. A more refined understanding of these mechanisms may facilitate the development of personalized, non-pharmacological strategies with the potential to improve functional outcomes and quality of life in individuals affected by NDDs.

## References

[1] World Health Organization. (2019). Risk reduction of cognitive decline and dementia: WHO guidelines. Geneva: World Health Organization. Available online at: https://www.who.int/news-room/fact-sheets/detail/dementia 31219687

[2] GBD 2021 Nervous System Disorders Collaborators. Global, regional, and national burden of disorders affecting the nervous system, 1990-2021: a systematic analysis for the global burden of disease study 2021. Lancet Neurol. (2024) 23:344–81. doi: 10.1016/S1474-4422(24)00038-3, 38493795 PMC10949203

[3] Selkoe DJ Hardy J. The amyloid hypothesis of Alzheimer's disease at 25 years. EMBO Mol Med. (2016) 8:595–608. doi: 10.15252/emmm.201606210, 27025652 PMC4888851

[4] Kalia LV Lang AE. Parkinson's disease. Lancet. (2015) 386:896–912. doi: 10.1016/S0140-6736(14)61393-3, 25904081

[5] Butterfield DA Halliwell B. Oxidative stress, dysfunctional glucose metabolism and Alzheimer disease. Nat Rev Neurosci. (2019) 20:148–60. doi: 10.1038/s41583-019-0132-6, 30737462 PMC9382875

[6] Trushina E McMurray CT. Oxidative stress and mitochondrial dysfunction in neurodegenerative diseases. Neuroscience. (2007) 145:1233–48. doi: 10.1016/j.neuroscience.2006.10.056, 17303344

[7] Dias V Junn E Mouradian MM. The role of oxidative stress in Parkinson's disease. J Parkinsons Dis. (2013) 3:461–91. doi: 10.3233/JPD-130230, 24252804 PMC4135313

[8] Kim J Cha YN Surh YJ. A protective role of nuclear factor-erythroid 2-related factor-2 (Nrf2) in inflammatory disorders. Mutat Res. (2010) 690:12–23. doi: 10.1016/j.mrfmmm.2009.09.007, 19799917

[9] Reddy NM Kleeberger SR Yamamoto M Kensler TW Scollick C Biswal S . Genetic dissection of the Nrf2-dependent redox signaling-regulated transcriptional programs of cell proliferation and cytoprotection. Physiol Genomics. (2007) 32:74–81. doi: 10.1152/physiolgenomics.00126.2007, 17895394

[10] Ma Q. Role of nrf2 in oxidative stress and toxicity. Annu Rev Pharmacol Toxicol. (2013) 53:401–26. doi: 10.1146/annurev-pharmtox-011112-140320, 23294312 PMC4680839

[11] Mizushima N Komatsu M. Autophagy: renovation of cells and tissues. Cell. (2011) 147:728–41. doi: 10.1016/j.cell.2011.10.026, 22078875

[12] Fernandes J Arida RM Gomez-Pinilla F. Physical exercise as an epigenetic modulator of brain plasticity and cognition. Neurosci Biobehav Rev. (2017) 80:443–56. doi: 10.1016/j.neubiorev.2017.06.012, 28666827 PMC5705447

[13] Cotman CW Berchtold NC. Exercise: a behavioral intervention to enhance brain health and plasticity. Trends Neurosci. (2002) 25:295–301. doi: 10.1016/S0166-2236(02)02143-4, 12086747

[14] Morais LH Schreiber HL t Mazmanian SK. The gut microbiota-brain axis in behaviour and brain disorders. Nat Rev Microbiol. (2021) 19:241–55. doi: 10.1038/s41579-020-00460-0 33093662

[15] Cryan JF O'Riordan KJ Cowan CS Sandhu KV Bastiaanssen TF Boehme M . The microbiota-gut-brain axis. Physiol Rev. (2019) 99:1877–2013. doi: 10.1152/physrev.00018.2018, 31460832

[16] Allen JM Mailing LJ Niemiro GM Moore R Cook MD White BA . Exercise alters gut microbiota composition and function in lean and obese humans. Med Sci Sports Exerc. (2018) 50:747–57. doi: 10.1249/MSS.0000000000001495, 29166320

[17] Mach N Fuster-Botella D. Endurance exercise and gut microbiota: a review. J Sport Health Sci. (2017) 6:179–97. doi: 10.1016/j.jshs.2016.05.001, 30356594 PMC6188999

[18] Cummings J Zhou Y Lee G Zhong K Fonseca J Cheng F. Alzheimer's disease drug development pipeline: 2023. Alzheimer's & Dementia. (2023) 9:e12385. doi: 10.1002/trc2.12385, 37251912 PMC10210334

[19] Poewe W Seppi K Tanner CM Halliday GM Brundin P Volkmann J . Parkinson disease. Nat Rev Dis Primers. (2017) 3:17013. doi: 10.1038/nrdp.2017.13 28332488

[20] Erickson KI Voss MW Prakash RS Basak C Szabo A Chaddock L . Exercise training increases size of hippocampus and improves memory. Proc Natl Acad Sci USA. (2011) 108:3017–22. doi: 10.1073/pnas.1015950108, 21282661 PMC3041121

[21] Marosi K Mattson MP. BDNF mediates adaptive brain and body responses to energetic challenges. Trends Endocrinol Metab. (2014) 25:89–98. doi: 10.1016/j.tem.2013.10.006, 24361004 PMC3915771

[22] Sandberg M Patil J D'Angelo B Weber SG Mallard C. NRF2-regulation in brain health and disease: implication of cerebral inflammation. Neuropharmacology. (2014) 79:298–306. doi: 10.1016/j.neuropharm.2013.11.004, 24262633 PMC3958930

[23] Mayer C Riera-Ponsati L Kauppinen S Klitgaard H Erler JT Hansen SN. Targeting the NRF2 pathway for disease modification in neurodegenerative diseases: mechanisms and therapeutic implications. Front Pharmacol. (2024) 15:1437939. doi: 10.3389/fphar.2024.1437939, 39119604 PMC11306042

[24] Massaad CA Klann E. Reactive oxygen species in the regulation of synaptic plasticity and memory. Antioxid Redox Signal. (2011) 14:2013–54. doi: 10.1089/ars.2010.3208, 20649473 PMC3078504

[25] Picard M McEwen BS. Psychological stress and mitochondria: a conceptual framework. Psychosom Med. (2018) 80:126–40. doi: 10.1097/PSY.0000000000000544, 29389735 PMC5901651

[26] Hood DA Memme JM Oliveira AN Triolo M. Maintenance of skeletal muscle mitochondria in health, exercise, and aging. Annu Rev Physiol. (2019) 81:19–41. doi: 10.1146/annurev-physiol-020518-114310, 30216742

[27] Youle RJ van der Bliek AM. Mitochondrial fission, fusion, and stress. Science. (2012) 337:1062–5. doi: 10.1126/science.1219855, 22936770 PMC4762028

[28] Pickles S Vigié P Youle RJ. Mitophagy and quality control mechanisms in mitochondrial maintenance. Current Biology. (2018) 28:R170–85. doi: 10.1016/j.cub.2018.01.004, 29462587 PMC7255410

[29] Spencer JP. The impact of flavonoids on memory: physiological and molecular considerations. Chem Soc Rev. (2009) 38:1152–61. doi: 10.1039/b800422f, 19421586

[30] Gomes BAQ Silva JPB Romeiro CFR Dos Santos SM Rodrigues CA Gonçalves PR . Neuroprotective mechanisms of resveratrol in Alzheimer's disease: role of SIRT1. Oxidative Med Cell Longev. (2018) 2018:8152373. doi: 10.1155/2018/8152373, 30510627 PMC6232815

[31] Vauzour D Rodriguez-Mateos A Corona G Oruna-Concha MJ Spencer JP. Polyphenols and human health: prevention of disease and mechanisms of action. Nutrients. (2010) 2:1106–31. doi: 10.3390/nu2111106, 22254000 PMC3257622

[32] Ristow M Zarse K. How increased oxidative stress promotes longevity and metabolic health: the concept of mitochondrial hormesis (mitohormesis). Exp Gerontol. (2010) 45:410–8. doi: 10.1016/j.exger.2010.03.014, 20350594

[33] Gomez-Cabrera MC Domenech E Romagnoli M Arduini A Borras C Pallardo FV . Oral administration of vitamin C decreases muscle mitochondrial biogenesis and hampers training-induced adaptations in endurance performance. Am J Clin Nutr. (2008) 87:142–9. doi: 10.1093/ajcn/87.1.142, 18175748

[34] Paulsen G Cumming KT Holden G Hallén J Rønnestad BR Sveen O . Vitamin C and E supplementation hampers cellular adaptation to endurance training in humans: a double-blind, randomised, controlled trial. J Physiol. (2014) 592:1887–901. doi: 10.1113/jphysiol.2013.267419, 24492839 PMC4001759

[35] Merry TL Ristow M. Mitohormesis in exercise training. Free Radic Biol Med. (2016) 98:123–30. doi: 10.1016/j.freeradbiomed.2015.11.032, 26654757

[36] Niedzielska E Smaga I Gawlik M Moniczewski A Stankowicz P Pera J . Oxidative stress in neurodegenerative diseases. Mol Neurobiol. (2016) 53:4094–125. doi: 10.1007/s12035-015-9337-5, 26198567 PMC4937091

[37] Feng ZT Zhang YY Xue CX. Bioactive compounds and exercise in aging and neurodegeneration: mechanistic insights from the gut-brain-metabolic axis. Front Nutr. (2026) 13:1781176. doi: 10.3389/fnut.2026.1781176, 42027568 PMC13099315

[38] Lin MT Beal MF. Mitochondrial dysfunction and oxidative stress in neurodegenerative diseases. Nature. (2006) 443:787–95. doi: 10.1038/nature05292, 17051205

[39] Block ML Zecca L Hong JS. Microglia-mediated neurotoxicity: uncovering the molecular mechanisms. Nat Rev Neurosci. (2007) 8:57–69. doi: 10.1038/nrn2038, 17180163

[40] Sayre LM Perry G Smith MA. Oxidative stress and neurotoxicity. Chem Res Toxicol. (2008) 21:172–88. doi: 10.1021/tx700210j, 18052107

[41] Markesbery WR. Oxidative stress hypothesis in Alzheimer's disease. Free Radic Biol Med. (1997) 23:134–47. doi: 10.1016/S0891-5849(96)00629-6, 9165306

[42] Bose A Beal MF. Mitochondrial dysfunction in Parkinson's disease. J Neurochem. (2016) 139:216–31. doi: 10.1111/jnc.13731, 27546335

[43] Ashleigh T Swerdlow RH Beal MF. The role of mitochondrial dysfunction in Alzheimer's disease pathogenesis. Alzheimers Dement. (2023) 19:333–42. doi: 10.1002/alz.12683, 35522844

[44] Kim J Kundu M Viollet B Guan KL. AMPK and mTOR regulate autophagy through direct phosphorylation of Ulk1. Nat Cell Biol. (2011) 13:132–41. doi: 10.1038/ncb2152, 21258367 PMC3987946

[45] Russell RC Yuan HX Guan KL. Autophagy regulation by nutrient signaling. Cell Res. (2014) 24:42–57. doi: 10.1038/cr.2013.166, 24343578 PMC3879708

[46] He C Klionsky DJ. Regulation mechanisms and signaling pathways of autophagy. Annu Rev Genet. (2009) 43:67–93. doi: 10.1146/annurev-genet-102808-114910, 19653858 PMC2831538

[47] Klionsky DJ Abdelmohsen K Abe A Abedin MJ Abeliovich H Acevedo Arozena A . Guidelines for the use and interpretation of assays for monitoring autophagy (3rd edition). Autophagy. (2016) 12:1–222. doi: 10.1080/15548627.2015.1100356, 26799652 PMC4835977

[48] Moon HY Praag HV. Physical activity and brain plasticity. J Exerc Nutr Biochem. (2019) 23:23–5. doi: 10.20463/jenb.2019.0027, 32018342 PMC7004567

[49] Liu-Ambrose T Donaldson MG. Exercise and cognition in older adults: is there a role for resistance training programmes? Br J Sports Med. (2009) 43:25–7. doi: 10.1136/bjsm.2008.055616, 19019904 PMC5298919

[50] Costigan SA Eather N Plotnikoff RC Taaffe DR Lubans DR. High-intensity interval training for improving health-related fitness in adolescents: a systematic review and meta-analysis. Br J Sports Med. (2015) 49:1253–61. doi: 10.1136/bjsports-2014-094490, 26089322

[51] Zhao JL Jiang WT Wang X Cai ZD Liu ZH Liu GR. Exercise, brain plasticity, and depression. CNS Neurosci Ther. (2020) 26:885–95. doi: 10.1111/cns.13385, 32491278 PMC7415205

[52] Pedersen BK Saltin B. Exercise as medicine - evidence for prescribing exercise as therapy in 26 different chronic diseases. Scand J Med Sci Sports. (2015) 25:1–72. doi: 10.1111/sms.12581, 26606383

[53] Carr AC Frei B. Toward a new recommended dietary allowance for vitamin C based on antioxidant and health effects in humans. Am J Clin Nutr. (1999) 69:1086–107. doi: 10.1093/ajcn/69.6.1086, 10357726

[54] Brigelius-Flohé R Traber MG. Vitamin E: function and metabolism. FASEB J. (1999) 13:1145–55. doi: 10.1096/fasebj.13.10.1145 10385606

[55] Tanumihardjo SA. Vitamin a and bone health: the balancing act. J Clinical Densitomet. (2013) 16:414–9. doi: 10.1016/j.jocd.2013.08.016, 24183637

[56] Rayman MP. Selenium and human health. Lancet. (2012) 379:1256–68. doi: 10.1016/S0140-6736(11)61452-9, 22381456

[57] Prasad AS. Zinc in human health: effect of zinc on immune cells. Mol Med. (2008) 14:353–7. doi: 10.2119/2008-00033.Prasad, 18385818 PMC2277319

[58] Barnham KJ Masters CL Bush AI. Neurodegenerative diseases and oxidative stress. Nat Rev Drug Discov. (2004) 3:205–14. doi: 10.1038/nrd1330, 15031734

[59] Crane FL. Biochemical functions of coenzyme Q10. J Am Coll Nutr. (2001) 20:591–8. doi: 10.1080/07315724.2001.10719063, 11771674

[60] Shay KP Moreau RF Smith EJ Smith AR Hagen TM. Alpha-lipoic acid as a dietary supplement: molecular mechanisms and therapeutic potential. Biochim Biophys Acta. (2009) 1790:1149–60. doi: 10.1016/j.bbagen.2009.07.026, 19664690 PMC2756298

[61] Spencer JP. Flavonoids and brain health: multiple effects underpinned by common mechanisms. Genes Nutr. (2009) 4:243–50. doi: 10.1007/s12263-009-0136-3, 19685255 PMC2775888

[62] Vauzour D Vafeiadou K Rodriguez-Mateos A Rendeiro C Spencer JP. The neuroprotective potential of flavonoids: a multiplicity of effects. Genes Nutr. (2008) 3:115–26. doi: 10.1007/s12263-008-0091-4, 18937002 PMC2593006

[63] Koblinsky ND Power KA Middleton L Ferland G Anderson ND. The role of the gut microbiome in diet and exercise effects on cognition: a review of the intervention literature. J Gerontol A Biol Sci Med Sci. (2023) 78:195–205. doi: 10.1093/gerona/glac166, 35977540 PMC9951060

[64] Fang P Kazmi SA Jameson KG Hsiao EY. The microbiome as a modifier of neurodegenerative disease risk. Cell Host Microbe. (2020) 28:201–22. doi: 10.1016/j.chom.2020.06.008, 32791113 PMC7430034

[65] Delgado-Peraza F Nogueras-Ortiz C Simonsen AH Knight DD Yao PJ Goetzl EJ . Neuron-derived extracellular vesicles in blood reveal effects of exercise in Alzheimer's disease. Alzheimer's Res Ther. (2023) 15:156. doi: 10.1186/s13195-023-01303-9 37730689 PMC10510190

[66] Stein AM Coelho FGM Vital-Silva TM Rueda AV Pereira JR Deslandes AC . Aerobic training and circulating neurotrophins in Alzheimer's disease patients: a controlled trial. Exp Aging Res. (2023) 49:1–17. doi: 10.1080/0361073x.2022.2048586 35253623

[67] Marlatt MW Potter MC Bayer TA van Praag H Lucassen PJ. Prolonged running, not fluoxetine treatment, increases neurogenesis, but does not alter neuropathology, in the 3xTg mouse model of Alzheimer's disease. Curr Top Behav Neurosci. (2013) 15:313–40. doi: 10.1007/7854_2012_237, 23670818 PMC4554490

[68] Kirkbir F Atasoy T Khodadadai D Sajedi H Keskin O Babaie M. Fasted-state aerobic exercise enhances cognition and hippocampal BDNF signaling in an Alzheimer's disease rat model. Neurochem Res. (2025) 51:18. doi: 10.1007/s11064-025-04637-y, 41420669

[69] Belviranlı M Okudan N Sezer T. Exercise upregulates Mitsugumin 53 and ameliorates behavioral deficits and mitochondrial biogenesis in a sporadic Alzheimer's disease model in rats. Arch Gerontol Geriatr. (2026) 141:106078. doi: 10.1016/j.archger.2025.106078, 41202432

[70] Lopes EDS Coelho FGM Tribess S Catarino JDS Ferreira BN Reis MM . Benefits of multimodal exercise intervention for BDNF and cytokines levels, cognitive function, and motor functionality in Alzheimer's disease: a preliminary study. Int J Environ Res Public Health. (2025) 22. doi: 10.3390/ijerph22081245, 40869831 PMC12386868

[71] Lee DY Im SC Kang NY Kim K. Analysis of effect of intensity of aerobic exercise on cognitive and motor functions and neurotrophic factor expression patterns in an Alzheimer's disease rat model. J Personalized Medicine. (2023) 13:13. doi: 10.3390/jpm13111622, 38003937 PMC10672300

[72] Fakhraei S Reza Almasi M Peeri M Gharakhanlou R. The effect of 4-week rehabilitation by aerobic exercise on hippocampus BDNF and TGF-β1 gene expressions inAβ 1-42-induced rat model of Alzheimer's disease. J Clin Neurosci. (2022) 95:106–11. doi: 10.1016/j.jocn.2021.11.027, 34929632

[73] Vasconcelos-Filho FSL da Rocha ESRC Martins JER Godinho WDN da Costa VV Ribeiro JKC . Neuroprotector effect of daily 8-minutes of high-intensity interval training in rat aβ(1-42) Alzheimer disease model. Curr Alzheimer Res. (2020) 17:1320–33. doi: 10.2174/1567205018666210218161856 33602092

[74] Kontush A Mann U Arlt S Ujeyl A Lührs C Müller-Thomsen T . Influence of vitamin E and C supplementation on lipoprotein oxidation in patients with Alzheimer's disease. Free Radic Biol Med. (2001) 31:345–54. doi: 10.1016/S0891-5849(01)00595-0, 11461772

[75] Arlt S Müller-Thomsen T Beisiegel U Kontush A. Effect of one-year vitamin C- and E-supplementation on cerebrospinal fluid oxidation parameters and clinical course in Alzheimer's disease. Neurochem Res. (2012) 37:2706–14. doi: 10.1007/s11064-012-0860-8 22878647

[76] Flinn JM Bozzelli PL Adlard PA Railey AM. Spatial memory deficits in a mouse model of late-onset Alzheimer's disease are caused by zinc supplementation and correlate with amyloid-beta levels. Front Aging Neurosci. (2014) 6:174. doi: 10.3389/fnagi.2014.00174, 25374537 PMC4205817

[77] Asadbegi M Komaki H Faraji N Taheri M Safari S Raoufi S . Effectiveness of coenzyme Q10 on learning and memory and synaptic plasticity impairment in an aged aβ-induced rat model of Alzheimer's disease: a behavioral, biochemical, and electrophysiological study. Psychopharmacology. (2023) 240:951–67. doi: 10.1007/s00213-023-06338-2, 36811650

[78] Zhang YH Wang DW Xu SF Zhang S Fan YG Yang YY . Α-Lipoic acid improves abnormal behavior by mitigation of oxidative stress, inflammation, ferroptosis, and tauopathy in P301S tau transgenic mice. Redox Biol. (2018) 14:535–48. doi: 10.1016/j.redox.2017.11.001, 29126071 PMC5684493

[79] Safarzadeh E Ataei S Akbari M Abolhasani R Baziar M Asghariazar V . Quercetin ameliorates cognitive deficit, expression of amyloid precursor gene, and pro-inflammatory cytokines in an experimental models of Alzheimer's disease in Wistar rats. Exp Gerontol. (2024) 193:112466. doi: 10.1016/j.exger.2024.112466, 38821324

[80] Rezai-Zadeh K Arendash GW Hou H Fernandez F Jensen M Runfeldt M . Green tea epigallocatechin-3-gallate (EGCG) reduces beta-amyloid mediated cognitive impairment and modulates tau pathology in Alzheimer transgenic mice. Brain Res. (2008) 1214:177–87. doi: 10.1016/j.brainres.2008.02.107, 18457818

[81] Rezai-Zadeh K Shytle D Sun N Mori T Hou H Jeanniton D . Green tea epigallocatechin-3-gallate (EGCG) modulates amyloid precursor protein cleavage and reduces cerebral amyloidosis in Alzheimer transgenic mice. J Neurosci. (2005) 25:8807–14. doi: 10.1523/JNEUROSCI.1521-05.2005, 16177050 PMC6725500

[82] Kook SY Lee KM Kim Y Cha MY Kang S Baik SH . High-dose of vitamin C supplementation reduces amyloid plaque burden and ameliorates pathological changes in the brain of 5XFAD mice. Cell Death Dis. (2014) 5:e1083. doi: 10.1038/cddis.2014.26, 24577081 PMC3944243

[83] Salehi O Sheikholeslami-Vatani D Hosseini SA. Positive effects of HIIT and vitamin E on cognitive function, angiogenesis and NRF2 antioxidant pathway in male rats with Alzheimer's disease. Biogerontology. (2025) 26:129. doi: 10.1007/s10522-025-10274-3 40562949

[84] Farzi A Teymoor Davani A Seyed A Salehi O Mosallanezhad Z. The effect of eight weeks of aerobic training with vitamin C on some apoptotic markers in the hippocampus tissue of rats with Alzheimer's disease; an experimental study. Neurol Res. (2025) 47:77–86. doi: 10.1080/01616412.2024.2448624 39754544

[85] Hashemi SA Ghadimi Z Ghaedi H Hashemi A. The effects of eight weeks of aerobic training with vitamin C on the expression pathway of antioxidants in the hippocampus tissue of TMT induced Alzheimer's disease rats. Brain Res. (2024) 1822:148645. doi: 10.1016/j.brainres.2023.148645, 37871672

[86] Puoyan-Majd S Parnow A Rashno M Heidarimoghadam R Komaki A. The protective effects of high-intensity interval training combined with Q10 supplementation on learning and memory impairments in male rats with amyloid-β-induced Alzheimer's disease. J Alzheimer's Dis. (2024) 99:S67–s80. doi: 10.3233/JAD-230096, 37212117

[87] Puoyan-Majd S Parnow A Rashno M Heidarimoghadam R Komaki A. Effects of pretreatment with coenzyme Q10 (CoQ10) and high-intensity interval training (HIIT) on FNDC5, Irisin, and BDNF levels, and amyloid-Beta (aβ) plaque formation in the Hippocampus of aβ-induced Alzheimer's disease rats. CNS Neurosci Ther. (2025) 31:e70221. doi: 10.1111/cns.70221, 39957598 PMC11831071

[88] Cho JY Um HS Kang EB Cho IH Kim CH Cho JS . The combination of exercise training and alpha-lipoic acid treatment has therapeutic effects on the pathogenic phenotypes of Alzheimer's disease in NSE/APPsw-transgenic mice. Int J Mol Med. (22010) 25:337–46. doi: 10.3892/ijmm_00000350 20127037

[89] Abu-Elfotuh K Hussein FH Abbas AN Al-Rekabi MD Barghash SS Zaghlool SS . Melatonin and zinc supplements with physical and mental activities subside neurodegeneration and hepatorenal injury induced by aluminum chloride in rats: inclusion of GSK-3β-Wnt/β-catenin signaling pathway. Neurotoxicology. (2022) 91:69–83. doi: 10.1016/j.neuro.2022.05.002, 35526705

[90] Molaei A Hatami H Dehghan G Sadeghian R Khajehnasiri N. Synergistic effects of quercetin and regular exercise on the recovery of spatial memory and reduction of parameters of oxidative stress in animal model of Alzheimer's disease. EXCLI J. (2020) 19:596–612. doi: 10.17179/excli2019-2082, 32483406 PMC7257248

[91] Walker JM Klakotskaia D Ajit D Weisman GA Wood WG Sun GY . Beneficial effects of dietary EGCG and voluntary exercise on behavior in an Alzheimer's disease mouse model. J Alzheimer's Dis. (2015) 44:561–72. doi: 10.3233/jad-140981 25318545

[92] Tao X Wang L Gong W. Untargeted metabolomics reveals the mechanisms of luteolin and exercise combination treatment against cognitive impairments in AD mice through modulating autophagy. J Nutr Biochem. (2025) 145:110011. doi: 10.1016/j.jnutbio.2025.110011, 40541584

[93] Tao X Zhang R Wang L Li X Gong W. Luteolin and exercise combination therapy ameliorates amyloid-β1-42 oligomers-induced cognitive impairment in AD mice by mediating Neuroinflammation and autophagy. J Alzheimer's Dis. (2023) 92:195–208. doi: 10.3233/JAD-220904, 36710678

[94] Boopathi S Mendonca E Gandhi A Rady A Darwish NM Arokiyaraj S . Exploring the combined effect of exercise and Apigenin on Aluminium-induced neurotoxicity in zebrafish. Mol Neurobiol. (2024) 61:5320–36. doi: 10.1007/s12035-024-03913-2, 38191695

[95] Zhang Z Wu H Huang H. Epicatechin plus treadmill exercise are neuroprotective against moderate-stage amyloid precursor protein/Presenilin 1 mice. Pharmacogn Mag. (2016) 12:S139–46. doi: 10.4103/0973-1296.182174, 27279698 PMC4883070

[96] Schimidt HL Carrazoni GS Garcia A Izquierdo I Mello-Carpes PB Carpes FP. Strength training or green tea prevent memory deficits in a β-amyloid peptide-mediated Alzheimer's disease model. Exp Gerontol. (2021) 143:111186. doi: 10.1016/j.exger.2020.111186, 33279659

[97] Lorek K Chalimoniuk M Langfort J Mączewska J Królicki L Markowska K . 12-week aerobic interval training boosts neuroplasticity and motor function in Parkinson's disease: insights from BDNF, [(18)F]Fluorodopa PET/CT, and EEG. Neurorehabil Neural Repair. (2025) 39:867–82. doi: 10.1177/15459683251360729, 40792387

[98] Naser AA Dehkordi KJ Radhi MN Taghian F Chitsaz A. The effect of multimodal exercise on the levels of BDNF and GDNF in patients with Parkinson's disease. Int J Prev Med. (2025) 16:35. doi: 10.4103/ijpvm.ijpvm_353_24, 40520030 PMC12165611

[99] Harro CC Shoemaker MJ Coatney CM Lentine VE Lieffers LR Quigley JJ . Effects of nordic walking exercise on gait, motor/non-motor symptoms, and serum brain-derived neurotrophic factor in individuals with Parkinson's disease. Front Rehab Sci. (2022) 3:1010097. doi: 10.3389/fresc.2022.1010097, 36311206 PMC9614339

[100] Segura C Eraso M Bonilla J Mendivil CO Santiago G Useche N . Effect of a high-intensity tandem bicycle exercise program on clinical severity, functional magnetic resonance imaging, and plasma biomarkers in Parkinson's disease. Front Neurol. (2020) 11:656. doi: 10.3389/fneur.2020.00656, 32793096 PMC7393207

[101] Tang C Liu M Zhou Z Li H Yang C Yang L . Treadmill exercise alleviates cognition disorder by activating the FNDC5: dual role of integrin αV/β5 in Parkinson's disease. Int J Mol Sci. (2023) 24. doi: 10.3390/ijms24097830, 37175535 PMC10178565

[102] Kim SH Hwang L Jin JJ Ko IG Kim YB Yoon HS . Resistance exercise improves spatial learning ability through phosphorylation of 5′-adenosine monophosphate-activated protein kinase in Parkinson disease mice. Int Neurourol J. (2021) 25:S55–62. doi: 10.5213/inj.2142336.168, 34844387 PMC8654314

[103] O'Callaghan A Harvey M Houghton D Gray WK Weston KL Oates LL . Comparing the influence of exercise intensity on brain-derived neurotrophic factor serum levels in people with Parkinson's disease: a pilot study. Aging Clin Exp Res. (2020) 32:1731–8. doi: 10.1007/s40520-019-01353-w 31606860

[104] Harpham C Gunn H Marsden J Bescos-Garcia R Murgatroyd C Connolly L. Home-based high-intensity interval training for people with Parkinson's: a randomized, controlled, feasibility trial. Health Sci Rep. (2025) 8:e71024. doi: 10.1002/hsr2.71024, 40661734 PMC12257130

[105] Soke F Kocer B Fidan I Keskinoglu P Guclu-Gunduz A. Effects of task-oriented training combined with aerobic training on serum BDNF, GDNF, IGF-1, VEGF, TNF-α, and IL-1β levels in people with Parkinson's disease: a randomized controlled study. Exp Gerontol. (2021) 150:111384. doi: 10.1016/j.exger.2021.111384, 33965556

[106] Gomes ESA Van den Heuvel OA Rietberg MB De Groot V Hirsch MA Van de Berg WDJ . (HIIT-the track) high-intensity interval training for people with Parkinson's disease: individual response patterns of (non-)motor symptoms and blood-based biomarkers-a crossover single-case experimental design. Brain Sci. (2023) 13. doi: 10.3390/brainsci13060849, 37371330 PMC10296509

[107] Marie A Leroy J Darricau M Alfos S De Smedt-Peyrusse V Richard E . Preventive vitamin a supplementation improves striatal function in 6-hydroxydopamine hemiparkinsonian rats. Front Nutr. (2022) 9:811843. doi: 10.3389/fnut.2022.811843, 35178422 PMC8843942

[108] Müller T Büttner T Gholipour AF Kuhn W. Coenzyme Q10 supplementation provides mild symptomatic benefit in patients with Parkinson's disease. Neurosci Lett. (2003) 341:201–4. doi: 10.1016/S0304-3940(03)00185-X, 12697283

[109] Attia HN Maklad YA. Neuroprotective effects of coenzyme Q10 on paraquat-induced Parkinson's disease in experimental animals. Behav Pharmacol. (2018) 29:79–86. doi: 10.1097/FBP.0000000000000342, 28902670

[110] Xu Y Xie M Xue J Xiang L Li Y Xiao J . EGCG ameliorates neuronal and behavioral defects by remodeling gut microbiota and TotM expression in Drosophila models of Parkinson's disease. FASEB J. (2020) 34:5931–50. doi: 10.1096/fj.201903125RR, 32157731

[111] Zibaei Yekta Y Ranjbar R Habibi A Alizadeh A. The interactive effects of the aerobic exercise and vitamin E supplementation on Parkinson's rat model. Cell J. (2024) 26:285–92. doi: 10.22074/cellj.2024.2023457.1515, 39066593

[112] Vauzour D. Dietary polyphenols as modulators of brain functions: biological actions and molecular mechanisms underpinning their beneficial effects. Oxidative Med Cell Longev. (2012) 2012:914273. doi: 10.1155/2012/914273, 22701758 PMC3372091

[113] Caruso G Godos J Privitera A Lanza G Castellano S Chillemi A . Phenolic acids and prevention of cognitive decline: polyphenols with a neuroprotective role in cognitive disorders and Alzheimer's disease. Nutrients. (2022) 14. doi: 10.3390/nu14040819, 35215469 PMC8875888

[114] Ashok A Andrabi SS Mansoor S Kuang Y Kwon BK Labhasetwar V. Antioxidant therapy in oxidative stress-induced neurodegenerative diseases: role of nanoparticle-based drug delivery systems in clinical translation. Antioxidants (Basel, Switzerland). (2022) 11. doi: 10.3390/antiox11020408, 35204290 PMC8869281

[115] Abbott NJ Rönnbäck L Hansson E. Astrocyte-endothelial interactions at the blood-brain barrier. Nat Rev Neurosci. (2006) 7:41–53. doi: 10.1038/nrn1824, 16371949

[116] Banks WA. From blood-brain barrier to blood-brain interface: new opportunities for CNS drug delivery. Nat Rev Drug Discov. (2016) 15:275–92. doi: 10.1038/nrd.2015.21, 26794270

[117] Anand P Kunnumakkara AB Newman RA Aggarwal BB. Bioavailability of curcumin: problems and promises. Mol Pharm. (2007) 4:807–18. doi: 10.1021/mp700113r, 17999464

[118] Youdim KA Joseph JA. A possible emerging role of phytochemicals in improving age-related neurological dysfunctions: a multiplicity of effects. Free Radic Biol Med. (2001) 30:583–94. doi: 10.1016/S0891-5849(00)00510-4, 11295356

